# Deletion of the Stress Response Gene *DDR48* from *Histoplasma capsulatum* Increases Sensitivity to Oxidative Stress, Increases Susceptibility to Antifungals, and Decreases Fitness in Macrophages

**DOI:** 10.3390/jof7110981

**Published:** 2021-11-18

**Authors:** Logan T. Blancett, Kauri A. Runge, Gabriella M. Reyes, Lauren A. Kennedy, Sydney C. Jackson, Sarah E. Scheuermann, Mallory B. Harmon, Jamease C. Williams, Glenmore Shearer

**Affiliations:** 1 Center for Molecular and Cellular Biology, The University of Southern Mississippi, Hattiesburg, MS 39406, USA; kauri@thrupore.com (K.A.R.); gabriella.reyes24@gmail.com (G.M.R.); lkennedy1@umc.edu (L.A.K.); sydney.jackson@usm.edu (S.C.J.); sspence3@tulane.edu (S.E.S.); malloryharmon@yahoo.com (M.B.H.); williamsjamease@gmail.com (J.C.W.); glen.shearer@usm.edu (G.S.J.); 2 Division of Infectious Diseases, Department of Internal Medicine, University of Cincinnati College of Medicine, Cincinnati, OH 45267, USA; 3 ThruPore Technologies, Inc., New Castle, DE 19720, USA; 4 Department of Cell and Molecular Biology, University of Mississippi Medical Center, Jackson, MS 39216, USA; 5 Mississippi INBRE Research Scholars Program, Mississippi INBRE, The University of Southern Mississippi, Hattiesburg, MS 39406, USA; 6 High Containment Research Performance Core, Tulane National Primate Research Center, Covington, LA 70433, USA

**Keywords:** *Histoplasma*, *DDR48*, paraquat, hydrogen peroxide, amphotericin-B, ketoconazole, ergosterol

## Abstract

The stress response gene *DDR48* has been characterized in *Saccharomyces cerevisiae* and *Candida albicans* to be involved in combating various cellular stressors, from oxidative agents to antifungal compounds. Surprisingly, the biological function of *DDR48* has yet to be identified, though it is likely an important part of the stress response. To gain insight into its function, we characterized *DDR48* in the dimorphic fungal pathogen *Histoplasma capsulatum*. Transcriptional analyses showed preferential expression of *DDR48* in the mycelial phase. Induction of *DDR48* in *Histoplasma* yeasts developed after treatment with various cellular stress compounds. We generated a *ddr48∆* deletion mutant to further characterize *DDR48* function. Loss of *DDR48* alters the transcriptional profile of the oxidative stress response and membrane synthesis pathways. Treatment with ROS or antifungal compounds reduced survival of *ddr48∆* yeasts compared to controls, consistent with an aberrant cellular stress response. In addition, we infected RAW 264.7 macrophages with *DDR48*-expressing and *ddr48∆* yeasts and observed a 50% decrease in recovery of *ddr48∆* yeasts compared to wild-type yeasts. Loss of *DDR48* function results in numerous negative effects in *Histoplasma* yeasts, highlighting its role as a key player in the global sensing and response to cellular stress by fungi.

## 1. Introduction

The stress response gene *DDR48* is required to combat oxidative stress, antifungal drugs, and numerous other stressors encountered by fungi. An identifying feature of *DDR48* is that it contains multiple repeats of S-Y-G, which seem to be conserved between all fungal species [1,2]. The SYG motif has been characterized as a prion-like domain with low complexity sequence that exhibits RNA binding properties [3,4]. SYG motif-containing proteins have been identified as components of stress granules, which are unique protein/RNA aggregates involved in RNA quality control, such as maintaining the balance between translating RNAs and mRNA degradation [3,5,6]. *DDR48* was identified in stress granules in the pathogenic fungus *C. albicans* [7], and overexpression of *DDR48* in *S. cerevisiae* resulted in a ~60% decrease in global RNA accumulation [8]. These findings demonstrate that *DDR48* functions in RNA metabolism in fungi.

*DDR48* has been extensively characterized in *C. albicans*; however, its function remains unknown. *DDR48* is among a unique subset of genes that are induced by the presence of ketoconazole, amphotericin-B, and 5-fluorocytosine [9]. Interestingly, *DDR48* is highly expressed during in vivo infections as well and is required for detoxification of the potent reactive oxygen species (ROS) hydrogen peroxide [1,2,10,11,12]. A *DDR48* mutant was more susceptible to the UV-like damage exerted by 4-nitroquinoline-1-oxide (4NQO) [12]. Hromatka et al. performed a genomic DNA microarray on *C. albicans* after exposure to reactive nitrogen species (RNS) and found *DDR48* levels to be upregulated by ~2-fold [13]. *DDR48* expression increases in response to amino acid starvation as well, which is dependent upon the general amino acid biosynthesis transcriptional activator *GCN4* [14]. A *DDR48* mutant strain is more susceptible to killing by the antifungal compounds itraconazole, fluconazole, and ketoconazole when compared to a wild-type strain [15,16]. Another study performed in 2014 expounded on the implications of *DDR48′s* role in antifungal drug resistance, in which clinical isolates of *C. albicans* that were fluconazole-resistant exhibited significantly increased quantities of *DDR48* mRNAs than fluconazole-sensitive isolates [11]. Interestingly, *DDR48* was identified as one of the 50 most highly abundant proteins in *C. albicans* during stationary growth [17]. To gain insight into the function of *DDR48*, we sought to characterize it in the dimorphic fungal pathogen *Histoplasma capsulatum*.

*Histoplasma capsulatum* is the etiological agent of histoplasmosis, one of the leading endemic mycoses in the world [18,19]. In the United States, *Histoplasma* is found primarily in the Mississippi and Ohio River Valley regions, with roughly 80% of the population having been exposed to the fungus, and over 500,000 new cases diagnosed annually [20,21]. *Histoplasma* is a thermally dimorphic fungus, existing in two distinct, temperature-dependent, forms. At environmental temperatures (25 °C), the fungus grows as a multicellular, spore-producing mycelia [22]. When soil containing *Histoplasma* spores is disturbed, the fungal elements are aerosolized and inhaled into mammalian lungs. The increase in temperature (37 °C) within the host triggers a transcriptional growth program in *Histoplasma* that promotes a shift to unicellular, pathogenic yeasts [23,24,25]. A unique feature of *Histoplasma* is the ability to become an intracellular pathogen of phagocytes, thus shielding it from the host immune system and providing a permissive niche for replication and dissemination [26,27,28,29]. *Histoplasma* yeasts produce several key virulence factors to promote survival and establish residence within phagocytic cells. As such, investigating these virulence factors and their pathways of regulation is important in understanding *Histoplasma* pathogenesis.

In this study, we characterized *DDR48* in the clinically relevant pathogen *Histoplasma capsulatum*. Transcriptional analyses showed preferential expression of *DDR48* in the mycelial phase; *DDR48* was induced in *Histoplasma* yeasts that were treated with various cellular stress compounds. We generated a *ddr48∆* deletion mutant to characterize *DDR48* function. Loss of *DDR48* alters the transcriptional profile of the oxidative stress response and membrane synthesis pathways. Treatment of mutant *ddr48∆* yeasts with ROS or antifungal compounds reduced survival. In addition, mutant yeasts did not thrive as well in macrophages as compared to wild-type yeasts. Thus, the absence of *DDR48* perturbs the response by *H. capsulatum* to stressors.

## 2. Materials and Methods

### 2.1. Strains and Culture Conditions

All *H. capsulatum* strains used in this study were derived from G186AS, a kind gift from William Goldman at University of North Carolina, Chapel Hill, which are listed in Table 1. G186AS is an attenuated variant [30] of G186AR (ATCC 26029), which was used in all experiments in this study due to biosafety concerns, as the majority of experiments involved undergraduate research students. *H. capsulatum* was cultured in *Histoplasma* macrophage medium (HMM), a basal cell culture medium that supports *H. capsulatum* growth [31]. All cultures were supplemented with ampicillin (50 µg/mL) and streptomycin (100 µg/mL) to decrease the likelihood bacterial contamination over long periods of growth and/or multiple samplings. *H. capsulatum* strains auxotrophic for uracil were supplemented with uracil (100 µg/mL). *H. capsulatum* yeasts were grown at 37 °C with shaking at 200 rpm until mid/late log phase before performing experiments. Growth of *H. capsulatum* yeasts was determined by culture turbidity at 600 nm or colony forming-united (CFUs). For measurement of growth kinetics, *H. capsulatum* yeasts were grown to mid/late log phase (OD_600_ = 1.8–2.2) then diluted with pre-warmed, pre-aerated HMM to a final OD_600_ = 0.1 and incubated at 37 °C with shaking at 200 rpm for a duration of 7 days. Turbidity measurements were taken every 24 h in triplicate to assess growth. For experiments involving *Histoplasma* mycelia, fresh yeast cultures were passed at a 1:10 dilution into room temperature, pre-aerated HMM, and incubated for 5–7 days at 25 °C to promote yeast-to-mycelia dimorphism. The cultures were then passed once more at a 1:10 dilution and used for experiments on day 3.

### 2.2. Generation of the ddr48∆ Mutant and DDR48-Complemented Strains

The *ddr48∆* deletion strain was previously created in our laboratory using allelic replacement technology described previously [32]. All primers used are listed in Table S1. Briefly, two fragments of *DDR48* genomic DNA (gDNA) were PCR amplified. A 5′ region fragment (5′) yielded a 660 bp product containing 603 bp upstream of the *DDR48* open reading frame (ORF) and 57 bp of *DDR48* ORF using primers ddr48-5′-F and ddr48-5′-R. A 3′ region fragment (3′) yielded a 766 bp product containing 680 bp downstream of the *DDR48* ORF and 86 bp of *DDR48* ORF using primers ddr48-3′-F and ddr48-3′-R. ddr48-5′-R and ddr48-3′-F both contain sequence of the hygromycin phosphotransferase gene (*hph*) to enable fusion cloning. Fragment 1 and fragment 2 combined would delete ~85% of the *DDR48* ORF. *hph* was amplified from a plasmid containing the *hph* ORF, pAN7-1, to yield a 3100 bp product (*hph*) using primers hph1-F and hph1-R.

Fusion PCR was conducted to fuse 5′, *hph*, and 3′ PCR products together to yield the *DDR48* knockout construct. A volume of 1 µL of each PCR product was used as template for the fusion PCR reactions and added to a mixture containing all PCR reagents except primers. Fusion PCR #1 was run as follows: (1) 95 °C for 1 min followed by (2) 95 °C for 20 s, 55 °C for 2 min, and 68 °C for 5 min repeated for a total of 14 cycles. Next, the tube was incubated at 68 °C for 10 min and placed on ice. A volume of 1 µL of product from Fusion PCR #1 was used as template for Fusion PCR #2. Fusion PCR #2 was primed by nested primers nest1-F and nest1-R to yield a ~4400 bp product containing the *DDR48* knockout construct with flanking *EcoRV* restriction endonuclease sites on each end. The product of Fusion PCR #2 was cloned into pCR2.1 TOPO Vector (Invitrogen, Waltham, MA, USA) following manufacturer’s recommendations to yield pDDR48-KO. pDDR48-KO was digested with *EcoRV* (New England Biolabs, Ipswich, MA, USA) and the *DDR48* knockout fragment and ligated into the *MscI* site of the telomeric expression vector pRPU1 [33], generating pDC01. This process is schematically represented in Figure S1a.

pDC01 was digested with *PmeI* to expose the telomeric repeats. Linearized pDC01 was then electroporated as previously described [33] into *H. capsulatum* strain WU27. Genomic replacement of *DDR48* was conducted using a modified positive and negative selection system previously described [34,35]. Briefly, *H. capsulatum* yeasts containing pDC01 were grown on solid HMM without uracil for uracil selection. Colonies were streaked onto a fresh HMM plate without uracil and incubated at 37 °C until colonies appeared. Individual transformants were inoculated into 1 mL of liquid HMM without uracil in 15 mL tubes. A 20 gauge needle was used to make a small hole on the screw cap of each tube to allow oxygenation. Samples were incubated at 37 °C on a roller drum for 7 days to allow for the double crossover event to occur. 200 µL from each tube was streaked onto selection plates containing HMM supplemented with 50 µg/mL uracil, 1 mg/mL 5-fluoroorotic acid (5-FOA), and 125 µg/mL hygromycin B. The plates were incubated at 37 °C for 14 days to select for hygromycin resistance (hygromycin B) and loss of pDC01 (5-FOA + uracil). gDNA was extracted from individual colonies and screened for deletion of *DDR48*. The recombination process is schematically represented in Figure S1b.

For complementation of *DDR48*, the *DDR48* gene and 1000 bp upstream of the start codon (to encompass the *DDR48* promoter region) were PCR amplified from parent strain WU27 genomic DNA using primers DDR48Comp-F and DDR48Comp-R. The PCR product was then cloned into the *MscI* site of pRPU1, creating pLE05. The *DDR48*-expressing telomeric vector (pLB05) was then transformed into the *ddr48∆* strain by electroporation as described previously [33]. URA^+^ transformants were screened for *DDR48* gene expression and positive transformants were chosen and confirmed rescue of *DDR48* expression by qRT-PCR and Northern blot.

### 2.3. Nucleic Acid Extractions and Blotting

DNA and RNA were extracted from mid/late log phase *Histoplasma* yeasts as previously described [36]. Briefly, cells were harvested by centrifugation and re-suspended in RNA extraction buffer (0.1 M NaCH_3_COO, pH 5.0, 0.2 M NaCl, 0.2% *w*/*v* sodium dodecyl sulfate) or DNA extraction buffer (0.1 M TRIS–pH 8.0, 0.1 M EDTA, 0.25 M NaCl) along with 0.5 mm acid washed glass beads and phenol:chloroform (5:1). The nucleic acids were then liberated by ballistic disruption with a MP Fast Prep-24 vortexer (MP Biomedicals, Irvine, CA, USA) until roughly 80% of cells were lysed as determined by microscopy. The nucleic acids were precipitated from the aqueous fraction with 100% ethanol. The pellets were air dried and resuspended in TE buffer (10 mM TRIS-HCl–pH 8.0, 0.5 M EDTA) (DNA) or RNase-free water (RNA) and stored at −80 °C until use. Northern blotting was performed as previously described [32]. Probes for northern blotting were generated by PCR amplification of WU27 genomic DNA using primers DDR48probe-F and DDR48probe-R for *DDR48* and HHT1probe-F and HHT1probe-R for *HHT1* (Table S1).

### 2.4. Quantitative Real-Time PCR

Primer pairs for each gene target used in this study can be found in Table S1. All primers were pre-tested to ensure high PCR efficiency and the lack of hairpins and/or dimer formations. RNA was extracted per the protocol listed above. Extracted RNA was subjected to DNAse treatment prior to cDNA synthesis using TURBO DNA-free kit (Invitrogen, Waltham, MA, USA) per the manufacturer’s instructions. A total of 500 ng of RNA was then reverse-transcribed using the Maxima First Strand cDNA Synthesis Kit for qRT-PCR, with dsDNase (Thermo Fisher Scientific, Waltham, MA, USA) per the manufacture’s protocol. The cDNA was quantified via A260/A280 using a NanoDrop (Thermo Fisher Scientific, Waltham, MA, USA) spectrophotometer and diluted to a final concentration of 500 ng/μL with nuclease-free water. The diluted cDNA stocks were stored at 4 °C until use.

Quantitative, real-time PCR (qRT-PCR) was performed on triplicate samples containing 500 ng of reverse-transcribed RNA using the Maxima SYBR Green/ROX qPCR Master Mix (2X) (Thermo Fisher Scientific, Waltham, MA, USA). Each reaction contained 0.2 μM forward primer, 0.2 μM reverse primer, 12.5 μL 2X SYBR Green/ROX Master Mix, 500 ng of cDNA, and nuclease-free water to a total volume of 25 μL. To reduce pipetting errors, a master mix was assembled for each gene-specific primer set containing all reagents except cDNA and dispensed into each tube. A volume of 1 μL of cDNA was then individually added to each tube. The qRT-PCR reactions were performed on a CFX96 Touch™ Real-Time PCR Detection System (Bio-Rad Laboratories, Hercules, CA, USA) using the following conditions: 95 °C for 10 min followed by 40 cycles of 95 °C for 15 s, 60 °C for 30 s, 72 °C for 30 s. Integrity of each run was measured by melt-curve analysis. Relative expression was determined using the Livak method (2^−∆∆Ct^) [37] after normalizing to levels of the constitutively expressed house-keeping gene *Histone H3 (HHT1)* transcript.

### 2.5. Cellular Stress Challenge

For cellular stress challenges, 500 mL of *H. capsulatum* yeasts were grown to mid-log phase (OD_600_ = 1.8–2.2) in HMM at 37 °C (yeast) and mycelia were grown for 5 days at 25 °C (mycelia) and then split into 50 mL aliquots. The various cellular stressors were then added to their respective 50 mL aliquot at optimized concentrations. Optimized concentrations were defined as the amount of each compound in which no growth inhibition of the *ddr48∆* strain was observed in liquid HMM. The stressors used include 0.1 µM paraquat-superoxide anions; 2.5 mM hydrogen peroxide–peroxides; 0.1 µg/mL amphotericin-B–membrane disruption; 0.25 µg/mL ketoconazole–sterol synthesis inhibition; 50 µg/mL 5-fluorocytosine–DNA/RNA biosynthesis inhibition 1 mM methyl methanesulfonate–DNA damage, methylating agent; 0.25 µM 4-nitroquinoline-1-oxide–UV-like damage; and heat-shock at 42 °C. The cultures were then incubated at 37 °C or 25 °C, respectively, with shaking, and 10 mL samples were taken at 15, 30, and 60 min after treatment for gene expression analysis. Samples were then subjected to RNA extraction, DNase treatment, cDNA synthesis, and gene expression measurement, as described above. Data was normalized to samples taken before the addition of each respective stress reagent.

### 2.6. Susceptibility to Superoxides and Peroxides

Yeast phase *Histoplasma* cultures were grown to mid-log phase (OD_600_ = 1.8–2.2). The cultures were then diluted with pre-warmed, pre-aerated, HMM to an OD_600_ = 0.1. To generate the superoxide anions, a 20 mM stock of paraquat dichloride was used to bring 50 mL aliquots of the diluted *H. capsulatum* cultures, in triplicate, to a final concentration of 0.5, 0.75, and 1.0 μM, respectively. Immediately before performing the experiment, a 33% *v*/*v* hydrogen peroxide solution (Sigma-Aldrich, St. Louis, MO, USA) was diluted with 1X PBS to make a fresh 50 mM hydrogen peroxide stock solution. The 50 mM stock was then used to bring 50 mL aliquots of diluted *H. capsulatum* cultures, in triplicate, to a final concentration of 2.5 mM, 5.0 mM, and 7.5 mM, hydrogen peroxide, respectively. Controls were also prepared by taking triplicate 50 mL aliquots of diluted *H. capsulatum* cultures with no additions. The cultures were then incubated at 37 °C in a humidified chamber with shaking at 200 rpm. After 72 h of incubation, aliquots from each culture were diluted and 100 µL spread onto HMM plates. Colony forming units (CFUs) were calculated from the plates once visible colonies were present.

### 2.7. ROS Measurements

ROS were detected using the fluorogenic dye 2′,7′-dichlorofluorescin diacetate (DCFDA), which is deacetylated by cellular esterases to a non-fluorescent compound within the cell and later oxidized by ROS into 2′,7′-dichlorofluorescin (DCF). DCF is highly fluorescent and can be detected by flow cytometry [38]. To measure ROS levels, a fresh 5 mM working stock of DCFDA (Invitrogen, Waltham, MA, USA) for each experiment per the manufacturer’s instructions. A total of 1 × 10^7^ *H. capsulatum* yeasts per ml were prepared in 10 mL volumes of HMM in 15 mL conical tubes, in triplicate. Each tube was supplemented with paraquat at a final concentration of 0.1 µM or an equivalent volume of PBS. DCFDA was then added to all tubes at a final concentration of 10 µM and incubated in the dark at 37 °C for 60 min. The tubes were then washed three times with 1X PBS and re-suspended in FACS buffer (1X PBS + 1 mM EDTA, 0.05% NaN_3_, and 2% FBS) containing 100 µg/mL propidium iodide (PI) (Abcam, Cambridge, UK) and incubated for 10 min in the dark at 4 °C. The samples were then analyzed by flow cytometry on an Aurora Spectral Flow Cytometer (Cytek Biosciences, Fremont, CA, USA) and subsequently gated on singlets, live cells (PI^−^), and DCF. Analysis of flow cytometry data was analyzed in FlowJo software (BD, Franklin Lakes, NJ, USA). All flow cytometric data were acquired using equipment maintained by the Research Flow Cytometry Core in the Division of Rheumatology at Cincinnati Children’s Hospital Medical Center, Cincinnati, Ohio.

### 2.8. Protein Extraction

*Histoplasma capsulatum* yeast cultures were grown in liquid HMM at 37 °C with rotary shaking until they reached mid exponential/late exponential growth phase (OD_600_ = 1.8–2.2) before harvest. Five milliliter aliquots of each culture were placed into 15 mL conical tubes (Corning Inc., Corning, NY, USA) and supplemented with 5 μL (0.25 mg) of 50 mg/mL cycloheximide (Sigma). The tubes were then incubated at room temperature for 10 min and centrifuged at 500× *g* for 5 min. Next, the supernatant was removed, and the cells were washed twice with an equal volume of ice-cold water and re-suspended in 2 mL of ice-cold water. The cells were then equally divided between two 2 mL screw cap tubes and allowed to equilibrate to 4 °C on ice for 5 min before being centrifuged at 500× *g* for 5 min. Once the supernatant was removed 200 μL of glass beads, 600 μL of protein extraction buffer (60 mM Na_2_HPO_4_, 40 mM NaH_2_PO_4_, 10 mM KCl, and 1 mM MgSO_4_–pH 7.5), and 6 μL of fungal protease inhibitor cocktail (Thermo Fisher Scientific, Waltham, MA, USA) was added to each screw-cap tube. The tubes were allowed to equilibrate to 4 °C on ice for 5 min. Total protein was then liberated by ballistic disruption on a MP Fast Prep–24 sample preparation system (MP Biomedicals, Irvine, CA, USA) at 4.0 m/s in 20 s intervals followed by 1-min intervals of resting on ice for a total of 4 cycles. Each tube was supplemented with 15 μL of 20% sodium dodecyl sulfate (SDS) and allowed to incubate on ice for 5 min. The lysate was centrifuged at 10,000× *g* for 5 min at 4 °C to pellet any cellular debris. The supernatant containing soluble protein was then recovered and placed into a clean, pre-chilled 1.5 mL microcentrifuge tube and stored at −80 °C until use.

### 2.9. Catalase and Superoxide Dismutase (SOD) Assays

The superoxide dismutase (SOD) assay was performed using the Superoxide Dismutase Assay Kit (Cayman Chemical) per the manufacturer’s protocol. The hydrogen peroxide destruction assay was performed using a modified protocol previously described [39,40,41] to extrapolate relative catalase activity. In brief, 33% hydrogen peroxide (HPO) (Sigma-Aldrich, St. Louis, MO, USA) was diluted to a final concentration of 10 mM using phosphate buffer (PB) (137 mM NaCl, 10 mM Na_2_HPO_4_, 2.7 mM KCl, and 1.8 mM KH_2_HPO_4_–pH 7.4), added to a clean 100 mL glass bottle, and placed on ice, immediately before performing the experiment. Eight micrograms of total protein lysate from each *H. capsulatum* strain was diluted to a final volume of 200 μL using PB and allowed to equilibrate to 4 °C on ice. The reactions were assembled immediately before use by adding 800 μL of the 10 mM HPO solution to 200 μL *H. capsulatum* protein extract, mixing briefly so not to create bubbles, and placed into a 1 mL quartz cuvette (Sigma-Alrich, St. Louis, MO, USA). The quartz cuvette was then immediately placed into the cuvette holder of a microplate reader (Molecular Devices, San Jose, CA, USA) and absorbance at 240 nm and 600 nm was recorded every 5 s for a total of 5 min. The above methods were repeated for each protein extract examined right before reading the absorbance to ensure the linear phase of catalase activity occurred in the measured time frame of the assay. One unit (U) of catalase activity was defined as the amount required to catalyze the decomposition of 1 mmol of HPO per minute in a 0.05 M HPO solution at 25 °C. The equation ∆OD 240 nm (1 min) × 1000/43.6 × mg protein was used to calculate the specific catalase activity of each reaction in unit per mg of protein per min. Degradation rate was calculated by transforming the ∆OD 240 nm data points into molarity using a standard curve of HPO concentration versus ∆OD 240 nm to calculate the change in molarity per minute.

### 2.10. Susceptibility to Antifungal Agents

Susceptibility of *H. capsulatum* strains to the antifungal drugs amphotericin-B and ketoconazole was determined using a modified microplate-based growth assay optimized for *H. capsulatum* developed by Goughenour et al. 2015 [42]. Briefly, *Histoplasma* yeasts were grown to mid/late log (OD_600_ = 1.8–2.2) and diluted to 4.0 × 10^6^ yeasts/mL in a total volume of 10 mL pre-warmed 2X HMM and 50 μL aliquots were added to each well of a 96-well, untreated flat-bottomed microplate. Next, 50 μL of water or water supplemented with amphotericin-B or ketoconazole, respectively, at twice the desired concentration (two-fold dilutions from 32 μg/mL to 0.03 μg/mL final concentrations) was added to each well for a total volume equaling 100 μL. Each well was mixed gently using a multichannel micropipette by gently pipetting up and down for 10 s, to avoid creating bubbles. The lid was placed on the microplate and sealed with Blenderm™ (3M, Saint Paul, MN, USA) breathable tape. The plates were incubated for 72 h at 37 °C in a humidified chamber with twice daily aeration by incubating plates on a bench-top rocker for 30-min intervals every 12 h. The absorbance of each well at 600 nm was measured using a Synergy microplate reader (BioTek, Winooski, VT, USA) on the fourth day of the experiment. The measurements were then entered in to GraphPad Prism software and 50% inhibition values (IC_50_) were calculated by nonlinear regression.

### 2.11. In Vitro Macrophage Infections

RAW 264.7 macrophages (ATCC TIB-71) from *Mus musculus* were thawed from a frozen DMSO stock and added to a T-75 cell culture flask containing complete DMEM (C-DMEM; DMEM supplemented with 10% *v*/*v* FBS, 50 µg/mL ampicillin, and 100 µg/mL streptomycin) and incubated at 37 °C in a humidified incubator with 5% CO_2_ and 95% room air atmosphere for 24 h. Once the culture reached ~80% confluence, the macrophages were dissociated with 0.25% trypsin-EDTA (Gibco, Waltham, MA, USA), quantified by microscopy with a hemacytometer, and 5 × 10^4^ macrophages were seeded into each well of a 24-well tissue culture plate in 1 mL volumes. For assays that required macrophage stimulation, the macrophages were allowed to adhere to the culture plate for 20 min before murine, recombinant interferon-gamma (IFNγ) (Invitrogen, Waltham, MA, USA) was added to each well at 10 ng/mL and incubated at 37 °C for 24 h. For infection, *Histoplasma* yeasts were quantified via a hemacytometer and diluted in HMM-M (HMM + 10% FBS, 584 mg/L L-glutamine, and 3.7 g/L NaHCO_3_) to a multiplicity of infection (MOI) = 5:1. The plates were then incubated at 37 °C for 2 h to facilitate phagocytosis. Each well was then washed with 1X Dulbecco’s phosphate-buffered saline (DPBS) (2.7 mM KCl, 1.5 mM KH_2_PO_4_, 136.9 mM NaCl, 8.9 mM Na_2_HPO_4_•7H_2_O–pH 7.4) three times to remove any extracellular *H. capsulatum* yeasts. The wells were re-suspended in an equal volume of HMM-M and incubated at 37 °C for 24 h. The culture medium was then removed from each well, washed with DPBS three times, and an equal volume of water added to lyse macrophages. Each well also underwent mechanical lysing by scraping the bottom of the well with a sterile micropipette tip. Dilutions of each lysate were plated on to triplicate HMM plates and incubated at 37 °C until visible colonies appeared. Yeast survival was determined by recovery of viable colony forming units (CFUs).

### 2.12. Phagocytosis Assay

Phagocytosis assays were performed using a modified protocol from Cordero et al. [43]. A total of 1.5 × 10^5^ RAW 264.7 macrophages were seeded into wells of a 6-well tissue culture plate, each containing an 18 mm diameter poly-L-lysine coated glass coverslip. The macrophages were incubated for 24 h at 37 °C in a humidified incubator with 5% CO_2_ and 95% room air atmosphere to allowed adherence to the coverslip. *Histoplasma* yeast cultures were grown in HMM at 37 °C to mid-log growth phase before being labelled with 40 μg/mL of NHS Rhodamine (Thermo Fisher Scientific, Waltham, MA, USA) for 30 min at 25 °C. The cells were then washed with an equal volume of DPBS three times before re-suspending in an equal volume of pre-warmed HMM and quantified by a hemacytometer. For infection, the media was removed from each well of macrophages, washed with DPBS three times, and *H. capsulatum* cells were added to each well in pre-warmed HMM-M at an MOI = 5:1. The plates were incubated for two hours at 37 °C to facilitate phagocytosis of the Rhodamine-labelled *H. capsulatum* yeasts. The plates were then washed with DPBS three times, fixed for 15 min at 25 °C in 4% formaldehyde in DPBS, and washed three times. Each coverslip was then removed, placed onto a clean glass microscope slide containing Vecta-Shield hard set mounting medium (Vector Laboratories, Inc., Burlingame, CA, USA), and allowed to set for 10 min before being sealed with clear nail polish. The slides were allowed to dry horizontally in the dark overnight at 25 °C before being stored vertically long term in a slide box at 4 °C until microscopic analysis. Images were captured using a Zeiss 510 Meta confocal microscope (Carl Zeiss AG, Jena, Germany) using differential interfering contrast (DIC) and the 650 nm laser at 60X oil immersion magnification. At least 8 different images were obtained, each of a different field of view. The number of macrophages and the number of *H. capsulatum* yeasts within each were recorded. At least 200 macrophages were counted for each *H. capsulatum* strain being analyzed. Percent phagocytosis was determined by calculating the ratio of macrophages containing *H. capsulatum* yeasts divided by the total number of macrophages quantified. The phagocytic index was calculated as the average number of *H. capsulatum* yeasts inside each macrophage.

### 2.13. Statistical Analysis

All data are represented as individual data points and the mean ± standard deviation (SD). Data were analyzed by Students *t* test, one-way analysis of variance (ANOVA), or two-way ANOVA followed by Tukey’s multiple comparisons test or Sidak’s multiple comparisons test using GraphPad Prism v9 (GraphPad Software, San Diego, CA, USA). A *p*-value ≤ 0.05 was considered significant.

## 3. Results

### 3.1. Identification of DDR48 in H. capsulatum

**Figure 1:**
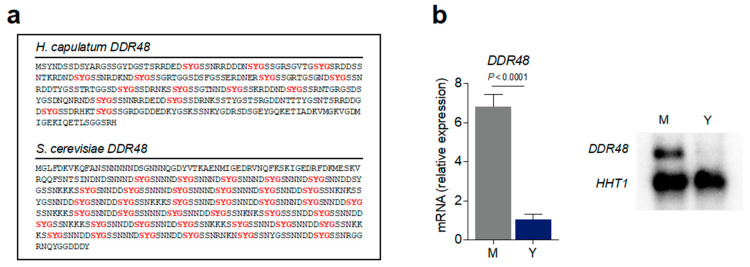
*DDR48* gene expression levels are enriched in *Histoplasma* mycelia. (**a**) amino acid sequence of *H. capsulatum* DDR48 (top) and *S. cerevisiae* DDR48 (bottom) showing SYG repeats (red). (**b**) Expression of *DDR48* in yeasts (Y) (blue bar) and mycelia (M) (grey bar) growth phases in liquid HMM measured by qRT-PCR (left) and representative northern blot (right) (*n* = 12) calculated relative to *HHT1* transcript levels. All data generated were performed on three technical replicates and at least two biological replicates. Data is represented as the mean ± standard deviation (SD). Statistical analyses were performed using Student’s *t* test.

The *H. capsulatum DDR48* gene (HCBG_02772) was originally isolated in our lab from a subtractive cDNA hybridization library enriched to identify transcripts whose expression was up-regulated in *Histoplasma* mycelia compared to *Histoplasma* yeasts [44]. Transcripts identified in this library were named as “mold-specific” plus the number corresponding to when it was identified chronologically (e.g., *mold-specific 8; MS8*). Using this naming system, *DDR48* was originally referred to as *mold-specific 95* (*MS95*). Reciprocal BLAST of *MS95* using the *Saccharomyces* Genome Database (SGD) found it to be an orthologue of the *S. cerevisiae DDR48* gene, sharing 48.6% identity. For continuity between fungal species in the literature, we updated the name from *MS95* to *DDR48*. Using the NCBI Conserved Protein Domain Family tool, we found *Histoplasma DDR48* to contain a conserved PTZ00110 (CDD) helicase domain belonging to the CL36512 domain superfamily. The PTZ00110 domain family consists of DEAD-box ATP-dependent RNA helicases involved in various aspects of RNA biosynthesis, maturation, and degradation [45,46]. Specifically, these RNA binding proteins contain a disordered amino acid region consisting of SYG repeats which are present in various numbers in fungal DDR48 proteins. The putative *Histoplasma* DDR48 amino acid sequence contains a total of 15 SYG repeats, compared to 36 SYG repeats found in *S. cerevisiae* DDR48 (Figure 1a). To validate the results of the cDNA library that *DDR48* preferentially expressed in *Hc* mycelia vs. yeasts, we compared *DDR48* transcript levels between the two morphotypes quantitatively via quantitative real-time PCR and qualitatively via Northern blot. As expected, *DDR48* was expressed at 6–7-fold higher levels in mycelia versus yeasts (Figure 1b), suggesting a possible mycelia-specific function.

To enable functional analysis of *DDR48* in *H. capsulatum*, we created a mutant strain lacking the *DDR48* gene. We replaced the *DDR48* gene with a constitutively expressed hygromycin resistance gene, *hph*. Roughly one kilobases of DNA sequence directly up-stream and down-stream of the *DDR48* gene flanked the *hph* gene to provide homologous sequences for recombination. The deletion strain *ddr48∆* was created by a homologous recombination event and the rare recombinants were screened by sequential positive and negative selections, schematically presented in (Figure S1). We next created a *DDR48*-complemented strain (*ddr48∆*/*DDR48*), which served to validate that any functional deficits observed in *ddr48∆* yeasts were due to the lack of *DDR48* and not off-target effects. A telomeric plasmid containing a functional copy of *DDR48*, its native promoter, and the *URA5* gene was inserted into the *ddr48∆* strain. The parental strain (WU27) lacks a functional *URA5* gene, which enables positive selection using uracil prototrophy [47]. We confirmed the absence of *DDR48* transcripts in the *ddr48∆* strain and successful complementation in the *ddr48∆/DDR48* strain by measuring *DDR48* and *HHT1* expression levels by qRT-PCR and northern blot. There were no *DDR48* transcripts detected in the *ddr48∆* strain and the complemented strain (*ddr48∆/DDR48)* restored *DDR48* expression to wildtype levels (Figure S2a). These results confirm successful generation of both a strain devoid of *DDR48* expression and a functional complemented strain restoring *DDR48* expression. To determine if there were any growth deficits between the *DDR48(+)*, *ddr48∆*, and *ddr48∆/DDR48* strains, we cultured yeasts in liquid HMM and observed their growth (Figure S2b). There were no detectable changes in growth rate of yeasts between the wild-type and mutant strains when cultured in HMM. To ensure that the chosen housekeeping gene, *HHT1*, was not fluctuating between wildtype and *ddr48∆* strains, we confirmed transcript levels of *HHT1* normalized to other common *Histoplasma* housekeeping genes: *ACT1*, *PFK1*, *TEF1*, and *GPD3* (Figure S2c). We found no significant differences in *HHT1* expression between wildtype, *ddr48∆*, and *ddr48∆/DDR48* yeasts.

### 3.2. DDR48 Is Constitutively Expressed in Histoplasma Mycelia and Inducible in Histoplasma Yeasts under Stress

**Figure 2:**
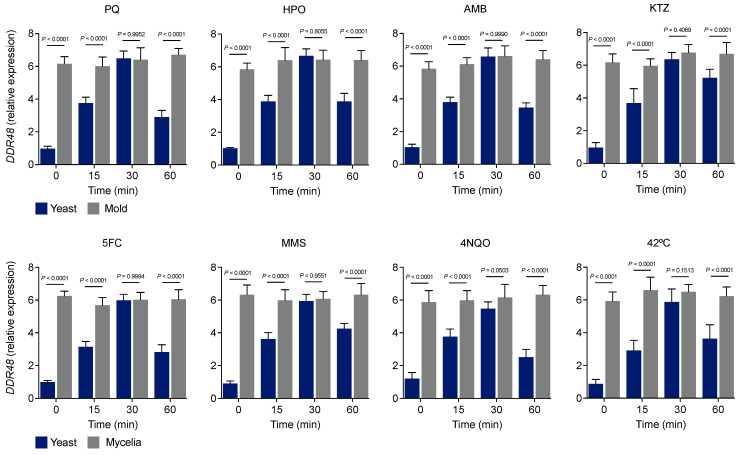
*DDR48* transcript levels are increased in *Histoplasma* yeasts subjected to cellular stressors. Expression of *DDR48* in yeast (blue bar) and mycelia (grey bar) growth phases measured by qRT-PCR (*n* = 9) at 0, 15, 30, and 60 min after the addition of various cellular stressors. The stressors include 0.1 µM paraquat (PQ)-superoxide anions, 2.5 mM hydrogen peroxide (HPO)-peroxides, 0.1 µg/mL amphotericin-B (AMB)-membrane disruption, 0.25 µg/mL ketoconazole (KTZ)-sterol synthesis inhibition, 50 µg/mL 5-fluorocytosine (5FC)-DNA/RNA biosynthesis inhibition, 1 mM methyl methanesulfonate (MMS)-DNA damage;methylating agent, 0.25 µM 4-nitroquinoline-1-oxide (4NQO)-UV-like damage, and heat-shock at 42 °C (HS). All data generated were performed on three technical replicates and at least two biological replicates. Data is represented as the mean ± standard deviation (SD). Statistical analyses were performed using one-way ANOVA with Tukey’s multiple comparisons test.

To enable functional analysis of *DDR48* in *H. capsulatum*, we created a mutant strain lacking the *DDR48* gene. In other fungi, *DDR48* is a known stress response gene that is upregulated in response to numerous cellular stressors [1,11,12,15,48,49,50]. We examined *DDR48* mRNA expression upon treatment of mycelia or yeasts with numerous stress-inducing compounds. Cells were grown in liquid culture to mid-late log phase, supplemented with each respective compound, and incubated at 37 °C (yeasts) or 25 °C (mycelia), respectively. In mycelial samples, *DDR48* was expressed constitutively and not altered by stress (Figure 2). In *Histoplasma* yeasts, however, *DDR48* expression was induced following exposure to each compound; expression peaked at 30 min post-treatment and returned to basal levels (Figure 2). Peak *DDR48* expression in yeasts mirrored basal *DDR48* expression in mycelia. Since mycelia grow at a much slower rate than yeasts, the lack of changes in *DDR48* transcript could simply be attributed to altered kinetics. To address this, we measured *DDR48* gene expression in mycelia at 3, 6, and 12 h post-treatment. *DDR48* expression remained at basal levels observed in the no treatment group (Figure S3). Thus, *DDR48* is constitutively expressed in mycelia, but inducible in *Histoplasma* yeasts under stress.

### 3.3. Loss of DDR48 Alters Intracellular Oxidative Stress Response and Increases Sensitivity to Oxidative Stress

**Figure 3:**
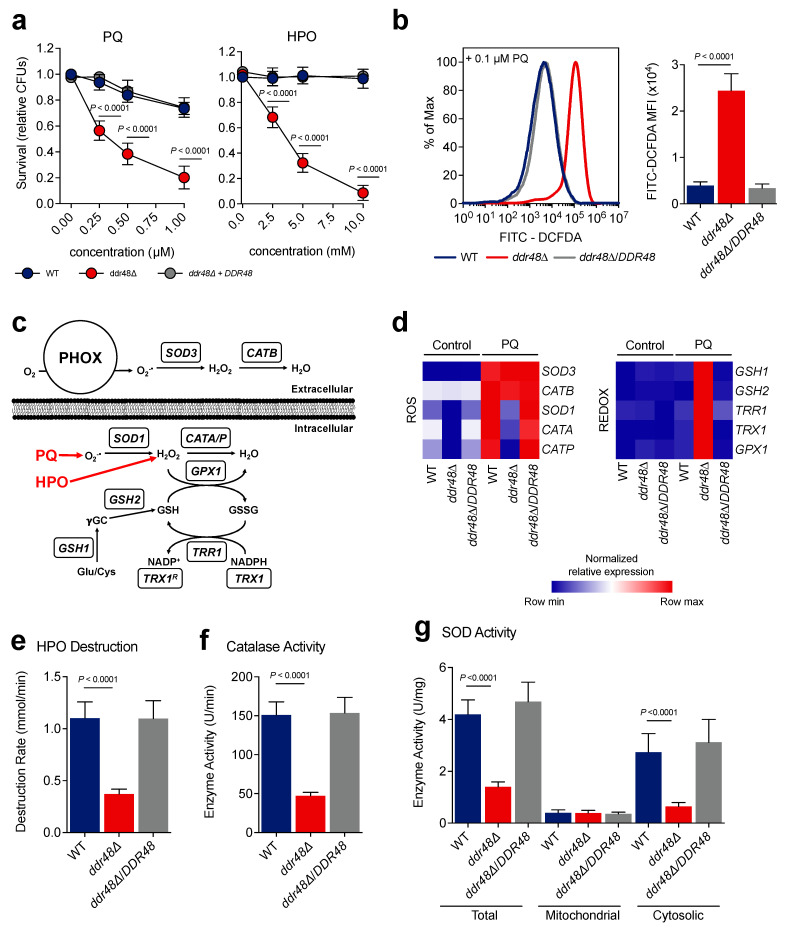
*ddr48∆* yeasts possess decreased antioxidant activity, resulting in increased ROS levels and killing. (**a**) Survival (relative CFUs) of wildtype (*DDR48(+)*), *ddr48∆*, and *ddr48∆/DDR48* strains measured (*n* = 8) after 4 days of growth in basal cell culture medium (HMM) supplemented with various concentrations of paraquat (PQ) or hydrogen peroxide (HPO). Survival was normalized to relative CFUs recovered from wildtype (*DDR48(+)*) yeasts with no treatment. (**b**) ROS levels measured (*n* = 8) in wildtype *(DDR48(+)*), *ddr48∆*, and *ddr48∆/DDR48* yeasts by DCFDA fluorescence and flow cytometry. Data is shown as a representative histogram (left) and median fluorescence intensity (MFI) measurements (right). (**c**) ROS/Antioxidant pathway utilized by *H. capsulatum* yeasts with representative genes and their function. The type of oxidant generated by paraquat (PQ) and hydrogen peroxide (HPO) are shown in bold red. (**d**) Transcriptional profile of wildtype (WT), *ddr48∆*, and *ddr48∆/DDR48* yeasts left untreated (Control) or treated with 0.1 µM PQ for 30 min (*n* = 9) measured by qRT-PCR relative to HHT1 levels. Expression of genes is presented as normalized row intensity (blue and red tiles) grouped by ROS or REDOX functions. (**e**) HPO destruction and (**f**) catalase activity of wildtype (WT), *ddr48∆*, and *ddr48∆/DDR48* protein extracts measured (*n* = 9) 10 s after the addition of 5 mM HPO. (**g**) Superoxide dismutase (SOD) enzymatic activity (*n* = 6) of mid-log phase wildtype (WT), *ddr48∆*, and *ddr48∆/DDR48* yeasts in HMM. Data is represented as the mean ± standard deviation (SD). Statistical analyses were performed using one-way ANOVA with Tukey’s multiple comparisons test.

Since expression of *DDR48* increases when treated with oxidative stress agents, we next assessed if *DDR48* plays a role in combatting oxidative stress in yeasts. To test if *DDR48* contributes to protection from reactive oxygen species (ROS), wildtype, *ddr48∆*, and *ddr48∆*/*DDR48* yeasts were challenged in vitro with various concentrations of paraquat (0.25 µM, 0.5 µM, and 0.75 µM) or hydrogen peroxide (2.5 mM, 5 mM, and 7.5 mM) to determine survival of each strain (colony forming units (CFUs)). Paraquat was used to generate superoxide [51]. Because superoxides are broken down to liberate peroxides, hydrogen peroxide was included as a ROS source. Wild-type, *DDR48*-expressing yeasts challenged with paraquat, or hydrogen peroxide exhibited greater than 80% survival (Figure 3a). However, only 20% of *ddr48∆* yeasts survive the highest concentration of paraquat and less than 5% survived the highest concentration of hydrogen peroxide (Figure 3a). Complementation of the *ddr48∆* deletion strain (*ddr48∆*/*DDR48*) restored survivability comparable to that observed in wild-type yeasts. To determine if the increased killing of *ddr48∆* yeasts with oxidative stress is due to less clearance of ROS, we treated wild-type, *ddr48∆*, and *ddr48∆*/*DDR48* yeasts with 0.1 µM paraquat and quantified ROS levels using the DCFDA assay via flow cytometry [38]. *ddr48∆* cells contained 3x more ROS than wild-type cells 1-h after treatment with paraquat (Figure 3b). Complementation of the *ddr48∆* mutant restored the ability to clear ROS generated by paraquat. These results demonstrate that *DDR48* contributes to protection of yeasts from both superoxide and peroxide species.

*Histoplasma* yeasts possess enzymes for detoxification of intracellular and extracellular reactive oxygen species (Figure 3c) [39,40,52,53]. To determine if the increased killing of *ddr48∆* yeasts by ROS is due to changes in gene expression of oxidative stress genes, we examined the transcriptional profile of the oxidative stress and redox pathways before and after treatment with 0.1 µM paraquat. Expression of the intracellular catalases (*CATA* and *CATP*) and cytosolic superoxide dismutase (*SOD1*) were decreased ~10-fold in *ddr48∆* yeasts before paraquat treatment, suggesting the loss of *DDR48* alters basal transcription of these genes (Figure 3d). In wild-type, *DDR48*-expressing yeasts, transcripts of *CATA*, *CATP*, and *SOD1* are upregulated after exposure to paraquat **(**Figure 3d). In paraquat-exposed *ddr48∆* yeasts, expression of *CATA, CATP,* and *SOD1* increased, albeit by the same fold-change but the magnitude differed. Complementation of the *ddr48∆* mutant restored gene expression to wild-type values. Expression of the extracellular catalase *CATB* and the extracellular superoxide dismutase *SOD3* was not affected by *DDR48*, as transcript levels were comparable between wild-type and *ddr48∆* yeasts before and after treatment (Figure 3d). These results suggest that *DDR48* is involved in combatting intracellular oxidative stress and not extracellular oxidative stress.

We next determined the transcriptional profile of the glutathione-dependent redox system before and after paraquat treatment, as it operates synergistically with ROS detoxification enzymes to maintain cellular redox homeostasis (Figure 3c) [54,55,56,57]. Gene expression of cytosolic thioredoxin reductase *TRR1*, cytosolic thioredoxin *TRX1*, glutathione peroxidase *GPX1*, glutamate-cysteine ligase *GSH1*, and glutathione synthetase *GSH2* before and after paraquat treatment was performed in wild-type, *ddr48∆*, and *ddr48∆/DDR48* yeasts. There were no significant changes in expression levels of wild-type yeasts after paraquat treatment; however, *TRR1*, *TRX1*, *GPX1*, *GSH1*, and *GSH2* were significantly upregulated in *ddr48Δ* yeasts after paraquat exposure (Figure 3d). Expression of *ddr48Δ/DDR48*-complemented yeasts remained unchanged, as in wild-type cells. To ensure that the changes in gene expression observed were not due to fluctuating *HHT1* mRNA levels, we confirmed transcript levels of *HHT1* normalized to *ACT1* before and after treatment with PQ or HPO. We found no significant differences in *HHT1* expression between wild-type and *ddr48∆* yeasts before or after treatment with PQ (Figure S4a) or HPO (Figure S4b). These results show that the glutathione-dependent oxidative stress system could be attempting to compensate for the loss of catalase and superoxide dismutase expression see in *ddr48Δ* yeasts.

To confirm that the changes in catalase and superoxide dismutase gene expression observed in the *ddr48∆* mutant corresponded with actual enzyme activity, we determined catalase and superoxide dismutase (SOD) enzymatic activity in wildtype, *ddr48Δ*, and *ddr48Δ/DDR48* yeasts. We measured catalase activity by absorption of light at 240 nm [41]. We optimized the assay for the concentration of *Histoplasma* protein and the time in which linear measurements could be taken (Figure S5). The rate of hydrogen peroxide decomposition decreased by 57% in *ddr48∆* yeasts compared to wild-type, *DDR48*-expressing yeasts (Figure 3e). Complementation of the *ddr48∆* strain returned the hydrogen peroxide decomposition rate to that resembling wildtype. The corresponding catalase activity of *ddr48∆* yeasts was 64% lower than wild-type and *ddr48∆/DDR48* strains (Figure 3f). We next subjected wildtype, *ddr48∆*, and *ddr48∆/DDR48* yeasts to 0.1 µM paraquat and measured superoxide dismutase activity levels from the corresponding cytoplasmic extracts. *ddr48∆* yeasts exhibited an 80% decrease in cytosolic and 64% decrease in total SOD enzyme activity compared to wild-type samples (Figure 3g). Interestingly, mitochondrial SOD activity was unaffected by the loss of *DDR48*. Cytosolic and total SOD activity levels were restored to wild-type levels in complemented *ddr48∆/DDR48* yeasts. These data demonstrate that the loss of *DDR48* results in decreased gene expression and corresponding decreases in enzymatic activity of intracellular superoxide dismutases and catalases, decreasing *Histoplasma* yeast’s response and survival against oxidative stress.

### 3.4. ddr48∆ Yeasts Are More Susceptible to Killing by Amphotericin-B and Ketoconazole

**Figure 4:**
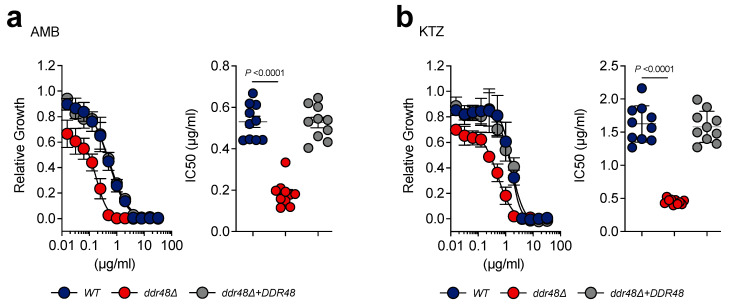
Loss of *DDR48* increases sensitivity to the antifungal drugs ketoconazole and amphotericin-B. Dose response curves (*n* = 10) of wildtype (WT), *ddr48∆*, and *ddr48∆*/*DDR48* yeasts for (**a**) amphotericin-B (AMB) and (**b**) ketoconazole (KTZ). IC_50_ values were determined by non-linear regression. Data is represented as the mean ± standard deviation (SD). Statistical analyses were performed using one-way ANOVA with Tukey’s multiple comparisons test.

Increased *DDR48* expression has been linked to antifungal drug resistance in clinical isolates of *C. albicans* [11]. We performed dose–response tests on wild-type, *ddr48∆*, and *ddr48∆/DDR48* strains to determine the concentrations for 50% inhibition (IC_50_) against amphotericin-B (polyene) and ketoconazole (azole) [58]. Wild-type yeasts treated with amphotericin-B showed an IC_50_ of 0.5296 ± 0.0858 µg/mL (mean ± standard deviation (SD)), whereas *ddr48∆* yeasts were 55% more susceptible to this antifungal with an IC_50_ of 0.1831 ± 0.0617 µg/mL (mean ± SD) (Figure 4a). *ddr48∆* yeasts (IC_50_ = 1.624 ± 0.2749 µg/mL (mean ± SD)) were 66% more susceptible to ketoconazole than wild-type yeasts (IC_50_ = 0.4514 ± 0.0397 µg/mL (mean ± SD)) (Figure 4b). No significant difference in the susceptibility to amphotericin-B or ketoconazole were found between wild-type and the complemented *ddr48∆/DDR48* strains. These data indicate that *DDR48* contributes to resistance to ketoconazole and amphotericin-B.

### 3.5. ddr48∆ Yeasts Exhibit an Aberrant Ergosterol Biosynthesis Transcriptional Program before and after Antifungal Drug Treatment

**Figure 5:**
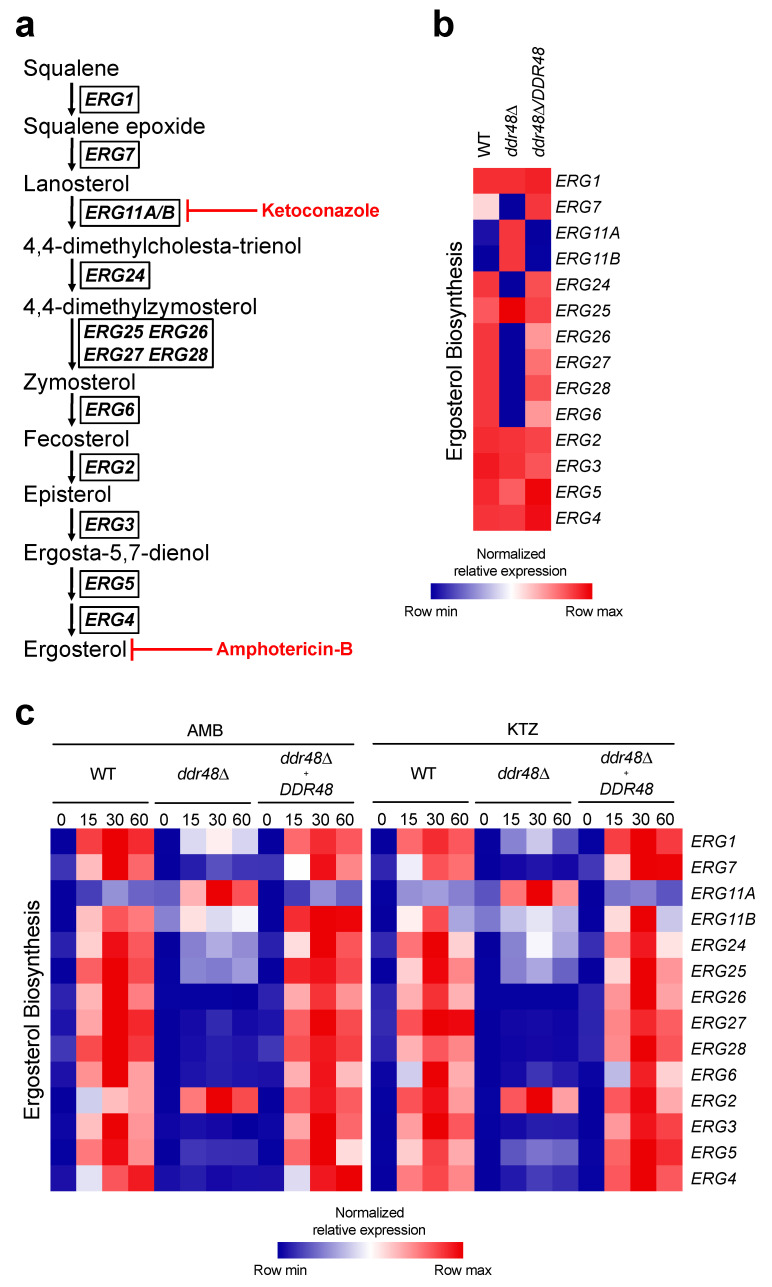
*DDR48*-Depleted *Histoplasma* Yeasts Contain an Aberrant Ergosterol Biosynthesis Pathway That is Exacerbated by Antifungal Treatment. (**a**) Ergosterol biosynthesis pathway utilized by *H. capsulatum* yeasts with representative genes and their function. The step at which each class of antifungals interferes with the pathway are shown in bold red. (**b**) Transcriptional profile of wildtype (WT), *ddr48∆*, and *ddr48∆*/*DDR48* yeasts grown in basal cell culture medium (HMM) and (**c**) left untreated (0) or treated with 0.1 µg/mL amphotericin-B (AMB; polyene) or 0.25 µg/mL ketoconazole (KTZ; azole) for 15, 30, and 60 min (*n* = 9) measured by qRT-PCR relative to *HHT1* levels. Expression of genes is presented as normalized row intensity (blue and red tiles) grouped by chronological step in the ergosterol biosynthesis pathway. All data generated were performed on three technical replicates and at least two biological replicates.

Amphotericin-B and ketoconazole exert their antifungal effects by targeting components of the ergosterol biosynthesis pathway as shown in (Figure 5a) [59,60,61]. Since deletion of *DDR48* affected susceptibility to amphotericin-B and ketoconazole, we examined the transcriptional profile of the ergosterol biosynthesis pathway before and 15, 30, and 60 min after treatment with these agents. Some ergosterol biosynthesis genes exhibited changes in basal expression between wild-type and *ddr48∆* yeasts before antifungal treatment (Figure 5b). *ERG11A* and *ERG11B* were upregulated by 5-fold and 10-fold, respectively, in the *ddr48∆* mutant strain. In contrast, *ERG7* (-3-fold)*, ERG24* (-10-fold)*, ERG26* (-3-fold)*, ERG27* (-7-fold)*, ERG28* (-16-fold)*,* and *ERG6* (-12-fold) were downregulated in *ddr48∆* yeasts (Figure 5b). These results suggest that *DDR48* affects basal transcription rates of 8 out of 14 (57%) of ergosterol biosynthesis genes. As expected, transcript levels of all the ergosterol biosynthesis genes tested increased upon treatment with amphotericin-B or ketoconazole in wildtype yeasts (Figure 5c).

Among *ddr48Δ* yeasts there were no significant changes in transcript levels of *ERG3, ERG4, ERG5, ERG11B,* and *ERG26* after treatment with amphotericin-B or ketoconazole (Figure 5c). Transcript levels of *ERG1*, *ERG6*, *ERG7, ERG24, ERG25, ERG27,* and *ERG28* increased in *ddr48Δ* yeasts, but did not reach magnitude of wildtype yeasts. *ERG11A* expression increased beyond wildtype after treatment, and no significant changes in *ERG2* expression were observed after treatment (Figure 5c). The complemented strain behaved similar to wildtype yeasts.

One possible explanation for these changes is that the housekeeping gene *HHT1* used to normalize expression is affected by the loss of *DDR48*. To ensure that the changes in gene expression were not caused by altered *HHT1* mRNA levels, we compared transcript levels of *HHT1* with *ACT1.* No significant differences in *HHT1* expression between wild-type and *ddr48∆* yeasts were observed after AMB (Figure S4c) or KTZ (Figure S4d) treatments. These data demonstrate that the loss of *DDR48* disrupts basal gene expressional levels of over half of ergosterol biosynthesis genes. The loss of *DDR48* disrupts the transcriptional program of the ergosterol biosynthesis pathway in responding to amphotericin-B and ketoconazole, leading to less resistance of yeasts to antifungal drugs.

### 3.6. ddr48∆ Yeasts Exhibit Decreased Survival in Macrophages

**Figure 6:**
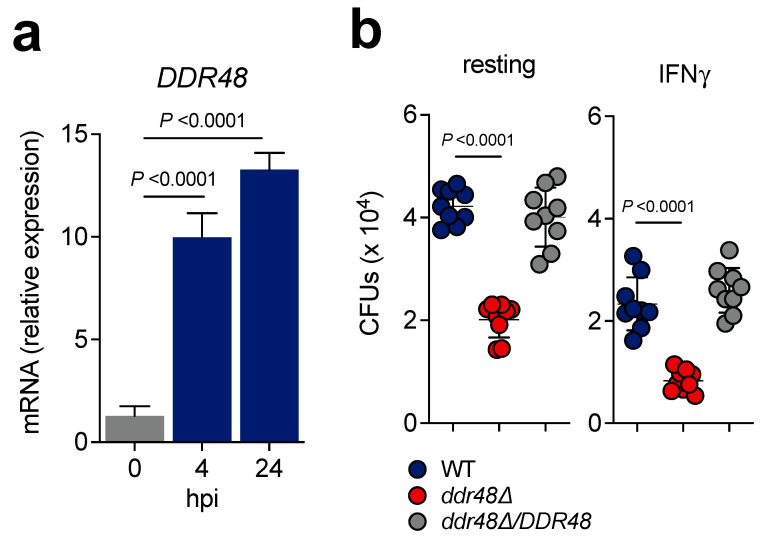
Deletion of *DDR48* decreases fitness of *Histoplasma* yeasts in macrophages. (**a**) RAW 264.7 macrophages were infected with wildtype yeasts (MOI 5:1) and *DDR48* mRNA levels were measured (*n* = 9) from uninfected (Ctrl) yeasts or from yeasts 4 h and 24 h post-infection by qRT-PCR and calculated relative to *HHT1* levels. (**b**) Resting (left) or stimulated (right; 10 ng/mL IFNγ) RAW 264.7 macrophages were infected with wildtype (WT), *ddr48∆*, or *ddr48∆*/*DDR48* yeasts (MOI 5:1) and in vitro macrophage growth of each strain was determined by lysis of macrophages and comparison of levels of viable yeasts (CFUs) in the lysate 24 h post-infection. All data generated were performed on three technical replicates and at least two biological replicates. Data is represented as the mean ± standard deviation (SD). Statistical analyses were performed using one-way ANOVA with Sidak’s multiple comparisons test.

Macrophages respond to *H. capsulatum* infection by initiating a respiratory burst that generates ROS [62]. Since *ddr48∆* yeasts are more sensitive to killing by ROS in vitro, we asked if deletion of *DDR48* affects intracellular survival within resting and stimulated RAW 264.7 macrophages. We first sought to determine if *DDR48* gene expression is upregulated once *Histoplasma* yeasts are phagocytosed by macrophages. At 4 h post-infection (hpi) *DDR48* expression was elevated by 10-fold and remained increased at 24 hpi (Figure 6a). We confirmed there were no changes in *HHT1* mRNA levels at 4 and 24 hpi (Figure S6a) Next, we infected resting and IFNγ-stimulated (10 ng/mL) RAW 264.7 macrophages with wild-type, *ddr48∆*, or *ddr48∆/DDR48* yeasts and examined *Histoplasma* CFUs at 24 hpi. *ddr48Δ* yeasts manifested a 50% decrease in CFUs in resting and IFNγ-stimulated macrophages compared to wild-type (Figure 6b). Recovery of the complemented *ddr48∆/DDR48* strain was similar to wildtype in resting and stimulated samples. IFNγ activation did increase macrophage killing of mutant and wildtype yeasts.

The changes in CFUs could be caused by differences in phagocytosis. Hence, we calculated the phagocytic index of wildtype, *ddr48∆,* and *ddr48∆/DDR48* yeasts and found no significant changes in the number of yeasts being phagocytized by each macrophage (Figure S6b). We found an average of 4 yeasts per macrophage. Next, we determined the percent of macrophages that contained *Histoplasma* yeasts of each strain and found no significant differences in phagocytosis between *DDR48*-expressing and *ddr48∆* yeasts (Figure S6c). These results affirm that *ddr48∆* yeasts are less fit (~50%) within macrophages compared to wild-type *Histoplasma* yeasts.

## 4. Discussion

In this study, we aimed to increase our understanding of the *H. capsulatum* *DDR48* gene, as little is known about *DDR48* in general, and there are no prior studies in *H. capsulatum*. *DDR48* was identified as a 315 amino acid protein containing fifteen repeats of the characteristic SYG amino acid sequence that is present in all identified *DDR48* amino acid sequences. The repetitive SYG motif is a putative RNA binding sequence believed to be involved in RNA regulation [3]. Given this information, we postulate that *DDR48* possesses RNA binding activity, providing a potential route in which it exerts its effects on the fungal stress response. Jin et al. identified *DDR48* as a component of processing bodies (*p*-bodies) and glycolytic bodies (G-bodies) in *C. albicans* [7], both of which are stress granules that function to maintain the balance between translating mRNAs and mRNA degradation under stress [63,64,65]. Each of these mRNA granules relies upon RNA binding proteins for proper structure and function [5,66], thus providing a link between *DDR48* and RNA metabolism. The *H. capsulatum DDR48* amino acid sequence contains a conserved domain belonging to a group of ATP-dependent RNA helicases (PTZ00110), which are known to be involved in RNA regulatory activities and localize to stress granules under stress [45,67]. PTZ00110 RNA helicases use ATP molecules to achieve their RNA un-winding activities, hence they also exhibit ATP hydrolysis [68]. A study in *S. cerevisiae* concluded that *DDR48* possesses ATP and GTP hydrolysis abilities [1], though no other studies have confirmed these results. These studies provide a foundation in which more investigations can begin to determine if *DDR48* has RNA binding properties and elucidate a possible role in RNA metabolism.

Although *DDR48* has been characterized as a mycelial phase-specific gene, our results demonstrated that *DDR48* is expressed in *Histoplasma* yeasts under cellular stress to levels observed in mycelia. Thirty minutes after treatment with hydrogen peroxide, *DDR48* expression was statistically similar (*p* > 0.05) to mycelial phase mRNA levels. A potential reason that *DDR48* is constitutively expressed in the mycelial phase is that the environmental conditions encountered by mycelia are variable, and depend on geographic location, nutrient availability, and weather conditions [69,70,71,72]. Constitutive expression in mycelia may enhance their ability to confront fluctuating environmental conditions.

Our studies are the first investigating the mode of action of *DDR48* in *H. capsulatum*. To gain more insight into *DDR48*, we subjected yeasts to a battery of cellular stressors and showed that *DDR48* was induced by all stressors used in this study, demonstrating the far-reaching effects of *DDR48* regarding fungal stress response. An interesting pattern emerged in which *DDR48* expression peaked 30 min post-treatment and began to decline thereafter. The expression pattern is similar to fungal heat shock proteins [73]. As an example, *Histoplasma* heat shock protein *HSP82* is induced transiently when the temperature is shifted to 34 °C [74]. Like *HSP82*, *DDR48* responded to heat shock, but at 42 °C. It is conceivable that *DDR48* acts like a heat shock protein since this family contributes to stress response and RNA metabolism [75,76,77]. On the other hand, RNA binding proteins that attach to and stabilize pre-existing mRNAs during the cellular stress response exhibit the same pattern of expression, peaking early on after stress treatment and quickly waning to basal levels [78,79,80,81].

We found that deletion of *DDR48* compromised *Histoplasma* yeast’s survival when treated with either superoxide or hydrogen peroxide. At the highest concentrations tested, *ddr48∆* yeasts were ~12-fold more susceptible to hydrogen peroxide and ~4-fold more susceptible to superoxides generated by paraquat. These results are consistent with experiments performed in *S. cerevisiae* and *C. albicans* [82,83]. In this work, we tracked gene expression of the intracellular and extracellular superoxide dismutases and catalases in wild-type and *ddr48∆* mutant strains. Youseff et al. previously showed that *H. capsulatum* contains an extracellular catalase and superoxide dismutase, *CATB* and *SOD3*, respectively, which are specialized to detoxify extracellular ROS produced by phagocytes [40,52,53]. When treated with oxidative stress, *CATB* and *SOD3* expression was unaffected by deletion of *DDR48*. This suggests that *DDR48* is required for *Histoplasma* survival of intracellular, but not exogenous oxidative stress. Our data did show that *DDR48* controls basal expression of the intracellular oxidative stress genes in addition to their response to oxidative stress. These findings suggest that *DDR48* has a function in RNA turnover. Overexpression of DDR48 in *S. cerevisiae* led to a ~60% decrease in global RNA accumulation [8], which conflicts with our findings. However, these two fungi are genetically very different. *Histoplasma* yeasts compensate for the loss of *DDR48* by activating alternative ROS detoxification pathways like the glutathione/thioredoxin system to aid in survival. This is consistent with studies in *S. cerevisiae* in which yeasts lacking the cytosolic thioredoxin system were more susceptible to killing by oxidative stress [54,84]. A potential reason for the glutathione/thioredoxin system to be upregulated in the *ddr48∆* strain is that paraquat induces ROS and glutathione/thioredoxin. However, the glutathione/thioredoxin system was unaltered in wild-type yeasts incubated with paraquat. This finding explains why the *ddr48∆* mutant is still able to proliferate, though at lower levels than wild-type, *DDR48*-expressing strains.

The antifungals fluconazole and itraconazole, in combination with amphotericin-B, are mainline treatments for infection with *Histoplasma capsulatum* [85]. One of the leading causes of treatment failure in HIV-positive patients with *H. capsulatum* infections is resistance to azole antifungals, thus new therapeutic approaches are needed to decrease relapse prevalence [86]. *ddr48Δ* yeasts are more susceptible to killing by ketoconazole and amphotericin-B. These results are consistent with studies in *C. albicans*; fluconazole-resistant isolates of *C. albicans* from patients exhibited higher expression of *DDR48*. Moreover, a strong correlation exists between overexpression of *DDR48* and azole resistance genes [11]. *DDR48* may exert its protective effects by modulating the ergosterol biosynthesis pathway. In this regard, the loss of *DDR48* impacted several genes of the ergosterol biosynthesis pathway.

*ddr48∆* yeasts were less fit within resting and activated macrophages, exhibiting a 50% reduction in survivability compared to wild-type yeasts. This was not caused by the exogenous ROS produced by phagocytes, as *CATB* and *SOD3*, the predominant enzymes that detoxify host-generated ROS were unaffected by deletion of *DDR48*. A probable explanation for the decreased survival of *ddr48∆* yeasts within macrophages is the intracellular ROS generated from cellular metabolism is accruing in cells, thus the *ddr48∆* yeasts are unable to cope with the harsh intramacrophage environment as efficiently as wild-type yeasts.

In summary, we have demonstrated that *H. capsulatum* relies on *DDR48* to adapt and recover to cellular stress. We have shown that it is needed for optimal survival in macrophages, response to oxidative stress, and response to antifungal drugs. More research into the function of *DDR48* will no doubt uncover more pathways in which *DDR48* is involved. Given the broad range of processes that are dependent upon *DDR48*, thought should be given to determining if it could be a successful therapeutic target for those predisposed or suffering from endemic fungal pathogens, as a pathogen that is not poised to adapt when exposed to various cellular stressors could tip the balance in favor of the host’s immune system and eventual elimination of the fungal pathogen.

## Figures and Tables

**Table 1:** *Histoplasma* strains used in this study.

Strain ^1^	Genotype ^2^	Other
		Designation
WU27	*ura5∆*	WT, *DDR48(+)*
USM10	*ura5∆ ddr48-3∆::hph*	*ddr48∆*
USM13	*ura5∆ ddr48-3∆::hph*/*pLE04 (URA5*, *DDR48)*	*ddr48∆*/*DDR48*

^1^ Strains were constructed in the *Histoplasma* G186AS background, a “smooth” colony variant [30] of G186A (ATCC 26029). ^2^ Gene designations: *hph*: hygromycin B phosphotransferase (hygromycin resistance). *URA5*: *Podospora anserina* orotate phosphoribosyltransferase. *DDR48*: stress response gene DDR48.

## Data Availability

The data that support the findings of this study are available from the corresponding author, L.T.B., upon reasonable request.

## Supplementary Materials

The following are available online at https://www.mdpi.com/article/10.3390/jof7110981/s1. Figure S1. Deletion, complementation, and confirmation of *HcDDR48*. Figure S2. Confirmation of *ddr48Δ*, growth characteristics, and qRT-PCR controls. Figure S3. *DDR48* transcript levels are constitutive in *Histoplasma* mycelia subjected to cellular stressors. Figure S4. *HHT1* gene expression remains constant in WT, *ddr48Δ*, and *ddr48Δ/DDR48* yeasts after exposure to cellular stress. Figure S5. Optimization of HPO decomposition assay. Figure S6. *HHT1* expression levels remain unchanged within macrophages. Table S1: Primers used in this study.
