# Supplementary material for: Deletion of the Stress Response Gene DDR48 from Histoplasma capsulatum Increases Sensitivity to Oxidative Stress, Increases Susceptibility to Antifungals, and Decreases Fitness in Macrophages
Source: J Fungi (Basel). 2021 Nov 18;7(11):981. doi: 10.3390/jof7110981 (PMC8617954; doi:10.3390/jof7110981)

Supplemental – Figures/Tables

jof-1437875 – Blancett et. Al.

Supplemental Figure S1

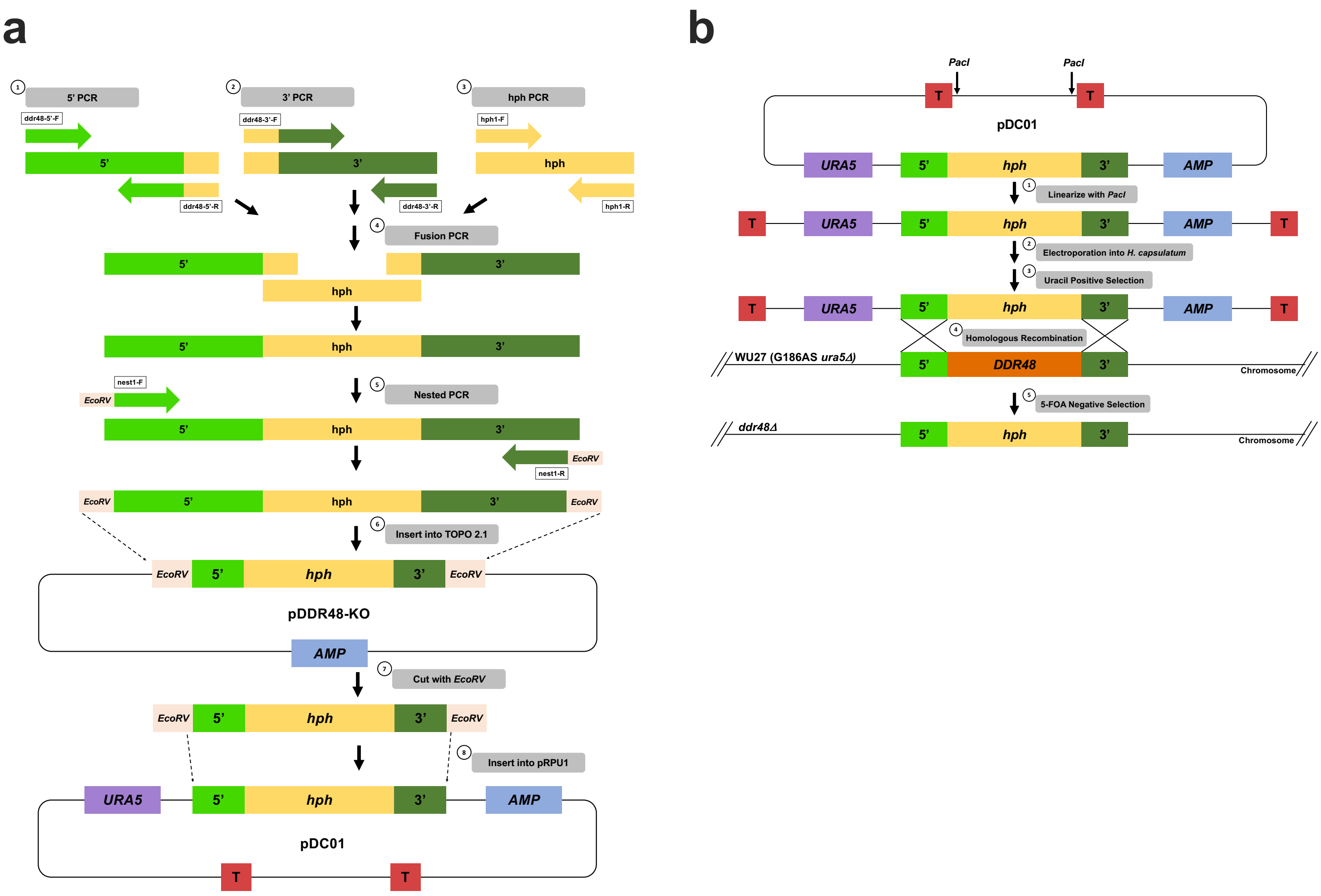

Sup. Fig. S1. Deletion, complementation, and confirmation of *HcDDR48*. a. Schematic diagram of PCRs to generate the *ddr48*Δ recombinant substrate. **b.** Schematic diagram of the plasmid features and steps performed for generation of the *H. capsulatum ddr48*Δ strain. T, telomere; URA5, orotidine monophosphate pyrophosphorylase; 5', 1000 bp upstream of DDR48 CDS, *hph*, hygromycin phosphotransferase, AMP, ampicillin resistance; 3', 1000 bp downstream of *DDR48* CDS.

Supplemental Figure S2

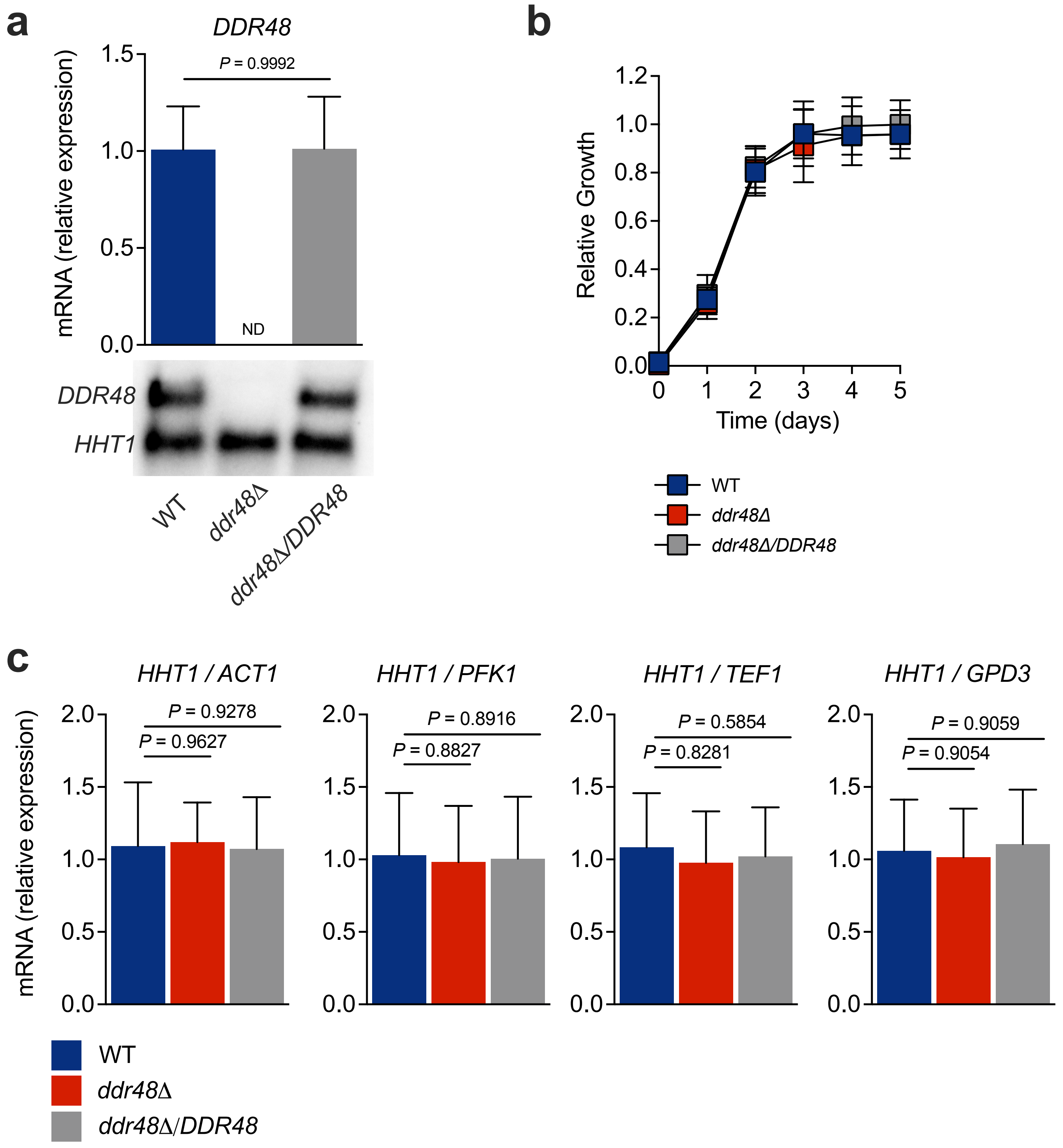

Sup. Fig. S2. Confirmation of *ddr48*Δ, growth characteristics, and qRT-PCR controls. a. *DDR48* mRNA levels were measured (n=6) in wildtype (*DDR48(+)*), *ddr48*Δ, and *ddr48*Δ/*DDR48* strains by qRT-PCR and calculated relative to *HHT1* levels. ND = no transcripts detected. **b.** Relative growth (OD 600 nm) of WT, *ddr48*Δ, and *ddr48*Δ/*DDR48* yeasts in HMM. **c.** Expression of *HHT1* relative to *ACT1*, *PFK1*, *TEF1*, and *GPD3*, measured by qRT-PCR (n = 12). Data is represented as mean ± standard deviation (SD). Statistical analyses were performed using 1-way ANOVA.

Supplemental Figure S3

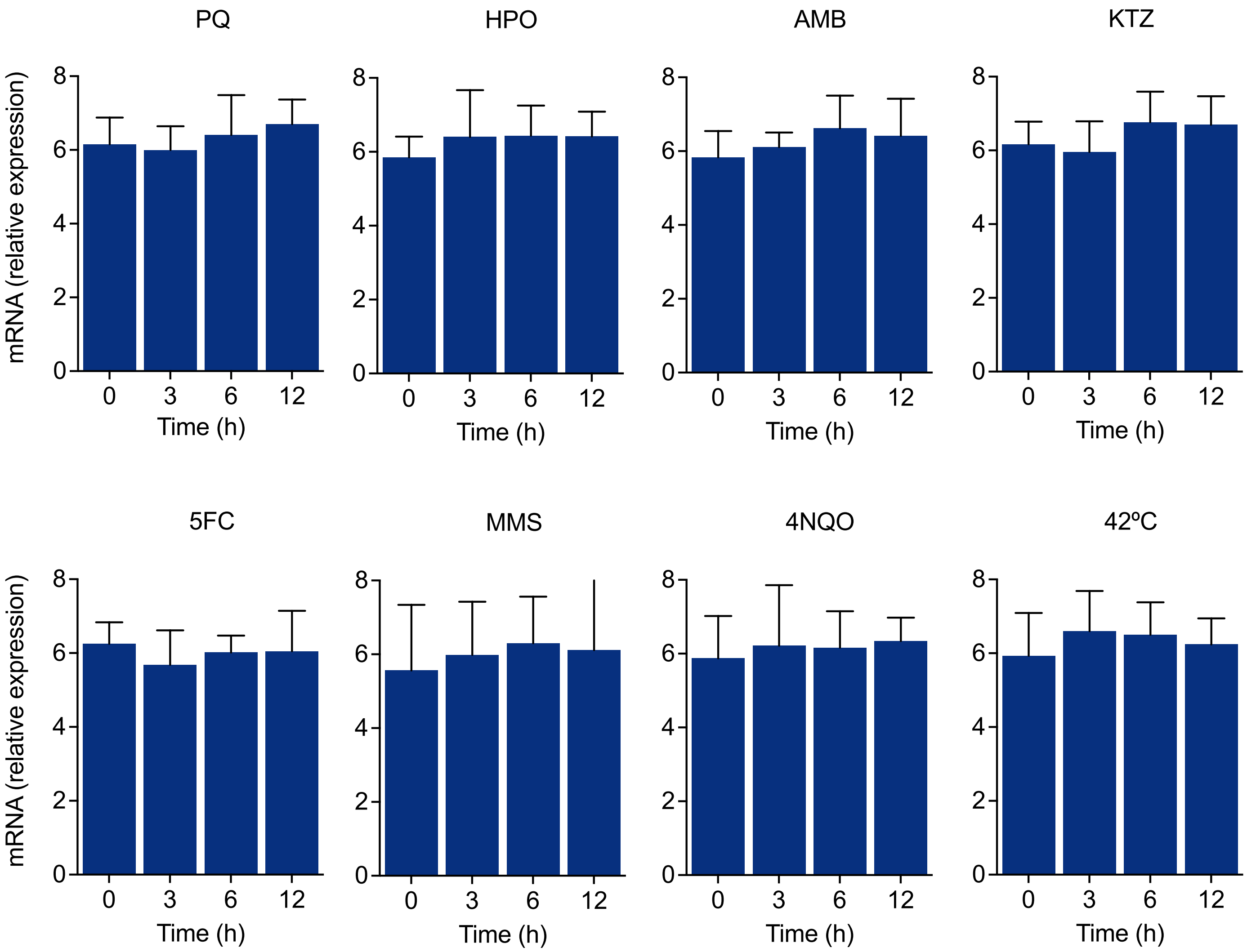

Supp. Fig. S 3. *DDR48* transcript levels are constitutive in *Histoplasma* mycelia subjected to cellular stressors. Expression of *DDR48* in mycelia growth phase measured by qRT-PCR (n=9) at 0, 3, 6, and 12 hours after the addition of various cellular stressors. The stressors include 0.1 µM paraquat (PQ); superoxide anions, 2.5 mM hydrogen peroxide (HPO); peroxides, 0.1 µg/ml amphotericin-B (AMB); membrane disruption, 0.25 µg/ml ketoconazole (KTZ); sterol synthesis inhibition, 50 µg/ml 5-fluorocytosine (5FC); DNA/RNA biosynthesis inhibition, 1 mM methyl methanesulfonate (MMS); DNA damage-methylating agent, 0.25 µM 4-nitroquinoline-1-oxide (4NQO); UV-like damage, and heat-shock at 42ºC (HS). All data generated were performed on three technical replicates and at least two biological replicates. Data is represented as mean ± standard deviation (SD). Statistical analyses were performed using 1-way ANOVA with Tukey's multiple comparisons test.

Supplemental Figure S4

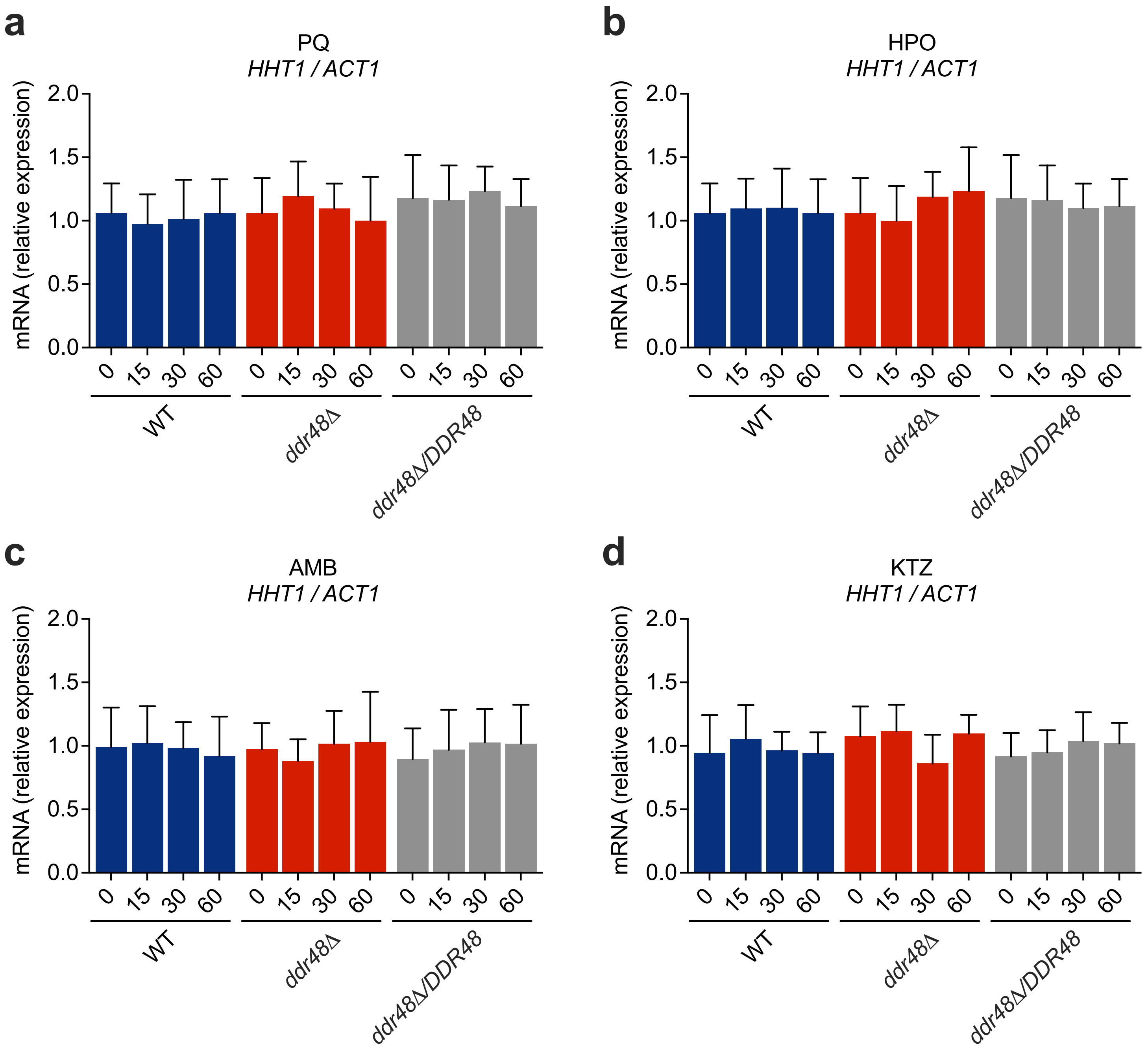

Sup. Fig. S4. *HHT1* gene expression remains constant in WT, *ddr48*Δ, and *ddr48*Δ/*DDR48* yeasts after exposure to cellular stress. Expression of *HHT1* measured by qRT-PCR (n=9) at 0, 15, 30, and 60 minutes post treatment with **a.** 0.1 μM PQ, **b.** 2.5 mM HPO, **c.** 0.1 μg/ml AMB, and **d.** 0.25 μg/ml KTZ. All data generated were performed on three technical replicates and at least two biological replicates. Data is represented as mean ± standard deviation (SD). Statistical analyses were performed using 1-way ANOVA with Tukey's multiple comparisons test.

Supplemental Figure S5

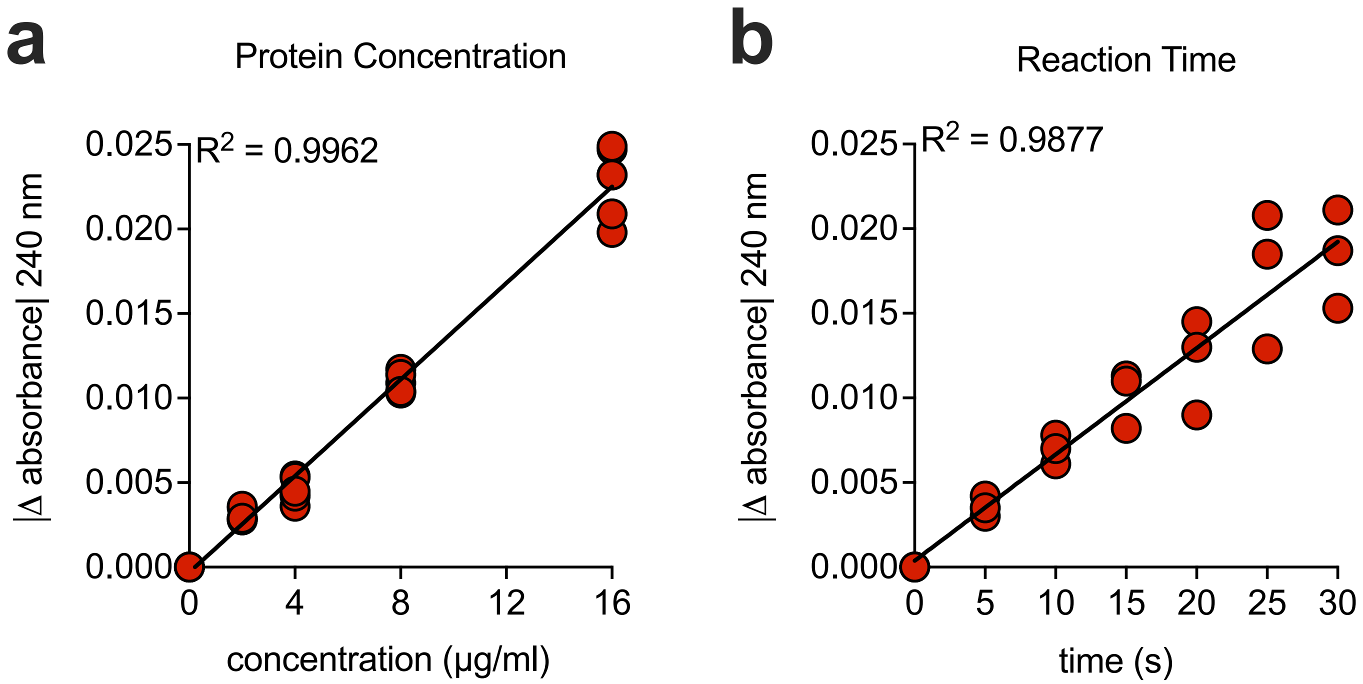

Sup. Fig. S5. Optimization of HPO decomposition assay. a. optimization of *Histoplasma* intracellular protein concentrations (n=4). Concentration of cellular lysates were determined by Bradford assay prior to assay. **b.** optimization of reaction time to determine linearity range using optimized protein concentration (8 μg/ml).

Supplemental Figure S6

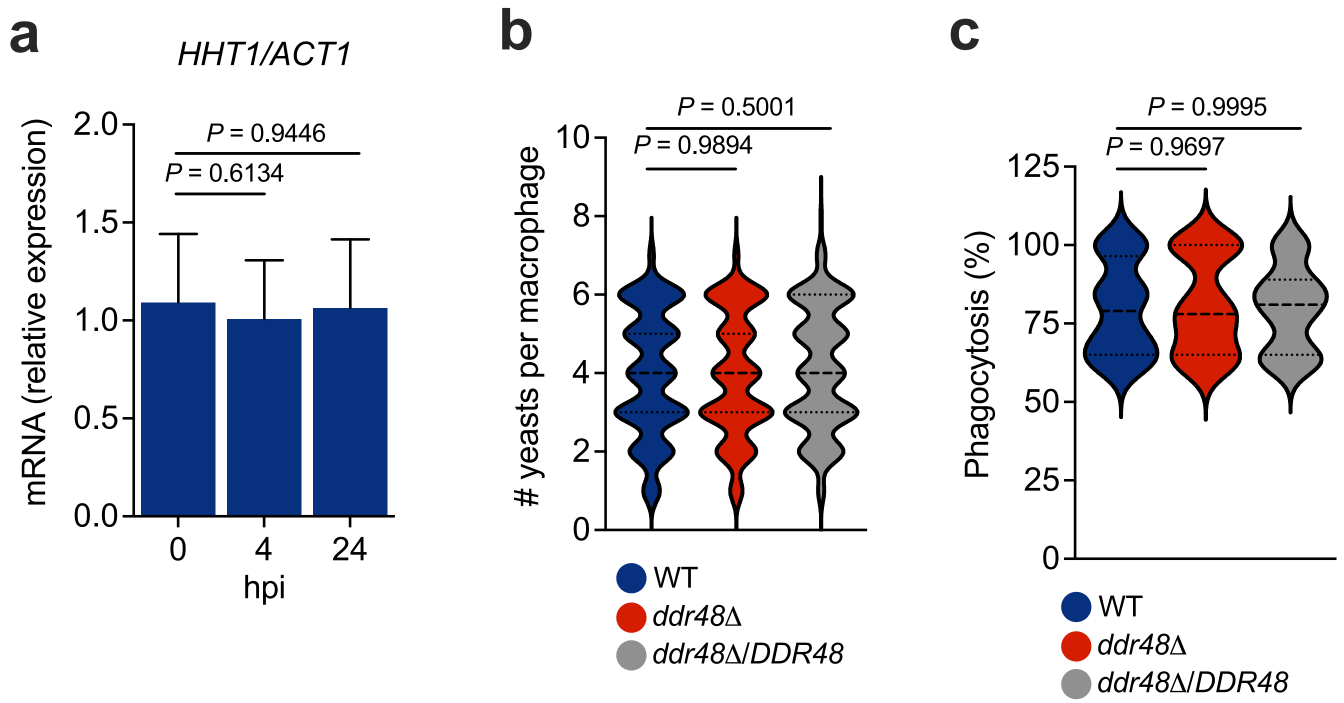

Sup. Fig. S6. *HHT1* expression levels remain unchanged within macrophages. a. Expression of *HHT1* relative to *ACT1* at 0, 4, and 24 hours post-infection (hpi) with RAW 264.7 macrophages at an MOI = 5:1 measured by qRT-PCR (n = 12). Macrophages were infected with wildtype (WT), *ddr48Δ*, or *ddr48Δ/DDR48* yeasts (MOI 5:1) and **b.** the phagocytic index and **c.** phagocytosis rate were determined. All data generated were performed on three technical replicates and at least two biological replicates. Data is represented as mean ± standard deviation (SD). Statistical analyses were performed using 1-way ANOVA with Sidak's multiple comparisons test.

Supplementary Table S1: Primers used in this study

Name	Sequence Direction[1] (5' to 3')		Use
	Forward	Reverse	
CATA	CCTCCCTATTATCCATCGATTTG	ACTGTATCGAAACTGCCTTTG	qPCR
ACT1	GATGGTGTGACCCACGTCGTTCCAATC	CGTTCAGGCGGGGCAATAATCTTAACCTTC	qPCR
CATB	CGGTGCTGGACAAATGTT	AGACCGTGAGTGAGTTGTA	qPCR
CATP	CCAACTTACACTGACTCCAATG	ATGGTGGTGATATCGCTGA	qPCR
DDR48	GACAATACTACCACCTATGGGTCTAA	CTTATCAGCGATGGTTTCCTTCTG	qPCR
ERG1	GTTTCAGTCATCCATTCGTCAAC	CGGAAGCTCTGCGTCTATAAG	qPCR
ERG11A[2]	TTCTTGGAACAAAAGGCAACG	CGAGGTTAGCCCGTATTTGAC	qPCR
ERG11B[2]	CTATGGAACCGACCCGTATAAG	TCGTTGCCCTTTATGCCTAG	qPCR
ERG2[2]	TTCAGCAACCACGGAAAC	CGCCATGCAGTATCGTAAA	qPCR
ERG24	CCTTCTACTCTTGTGCTTGATAC	AGTGGTAAGAAAGGTGTTGAAT	qPCR
ERG25[2]	GCAATAAAATCCCTAGCCTGAAG	TTTGATAAGTCATAGTCCACGGG	qPCR
ERG26	TATACAGAGACGAAGGCCCAA	GAGGACGAATGGAGAGGATTTG	qPCR
ERG27	GGAGGAAGATTGGTCCTATTG	ACTTTGACTGCCTGTGTAATG	qPCR
ERG3[2]	GGATTATGCCAAGCCCTTAC	CAGGACAGTCCAGATGTTAATG	qPCR
ERG4	GTACATCGTCTATCTGTTTGTTTAC	GCATATCCATACCACCCATC	qPCR
ERG5	CCACCATCTTCACCATCTTG	CGACGAACTTGTGGAAGAC	qPCR
ERG6	CTCTTACGCGACATTACTACAA	CCACAGCCTACATCAAGAAC	qPCR
ERG7	GGCACCTGTATGAACTACAC	GGAAGCAACCAGAGTTCAG	qPCR
GPD3	CATGCAAAACCGAGAAACCC	CCCACTCATTGTCGTACCAG	qPCR
GPX1	CTTCAGTGCGCTCCAGTAATAG	GGACAACCTTGCCTTTGAGA	qPCR
GSH1	GGAGAGAAGCCGCTGAAGAATGATCGC	GATGAAGAGGTGGGCGAAGTGCG	qPCR
GSH2	GGTTGGGGAAGATTATTGAAGAACTCAAGGAC	ATGAGGAGGAGATGGTGTTAAATTCCACTTGTT	qPCR
HHT1	TGGTAAGGTCCCTCGTAAGC	GGAGTTTGCGGATGAGGAG	qPCR
PFK1	GATTTGTGATTGAAGTGATGGGACGACATTGCG	GCGTTTGCCTCGTTTTCGATTCTGAGTGATAATTTC	qPCR
SOD1	CTTGTGGCGTCATTGGTATCACCACG	CCTCTCCTTCACAACTAAAGCACAGGTG	qPCR
SOD3	GGTTCTTTGACCCTGCAATAA	GTTACGTTAGGTGGGTTGTTAG	qPCR
SRB1[2]	GTAGCAGCCGAACAACATCTG	AATGAGACCTTGGGCGATACG	qPCR
TEF1	GCTCTGCTTGCTTTCACCCTTG	TCTCCTTGTTCCAGCCCTTGT	qPCR
TRR1	GATTACAAGTGCGGGATCTG	CCCATGTTACACATCTATCTTCTC	qPCR
TRX1	GACTCCATCGTCCAAACCA	GAAGGAGGTGCGGTCTTT	qPCR
ddr48-5'	GAGTCTCTCAATTGGATCCTTAATCAAGCGG	CTTCTCGGCGTTCTGGAGGTGATCTTACTCC	PCR *ddr48Δ*
ddr48-3'	CTTCCAGATCCATCGCTGATAAGGTAATGGGTAAAGTTG	CATACTTTGAAGAAATGGACGCACGAAGC	PCR *ddr48Δ*
hph1	CCTCCAGAACGCCGAGAAGAACTGGAGGGGT	ATCTGGAAGAGGTAAACCCGAAAAC	PCR *ddr48Δ*
nest1	AAGATATCCATCGCTGATATGCTTTTCCAGCTATTGAC	AAGATATCCACCTGGGCCCTCCTCTTCCCTCCC	PCR *ddr48Δ*
ddr48-probe	GATAGTTATGGCTCCTCCAACC	GAGTCGCTCCTATCTCCATACT	PCR probe
hht1-probe	GAGTCGCTCCTATCTCCATACT	AACAGCGCCAGTCATCTAAG	PCR probe

[1] Direction relative to mRNA transcript

[2] Sequences derived from DuBois & Smulian 2016.

DuBois, J.C.; Smulian, A.G. Sterol regulatory element binding protein (Srb1) is required for hypoxic adaptation and virulence in the dimorphic fungus Histoplasma capsulatum. *PLoS One* **2016**, *11*, e0163849

Supplemental – Full Blots
jof-1437875 – Blancett et. Al.

Figure 1

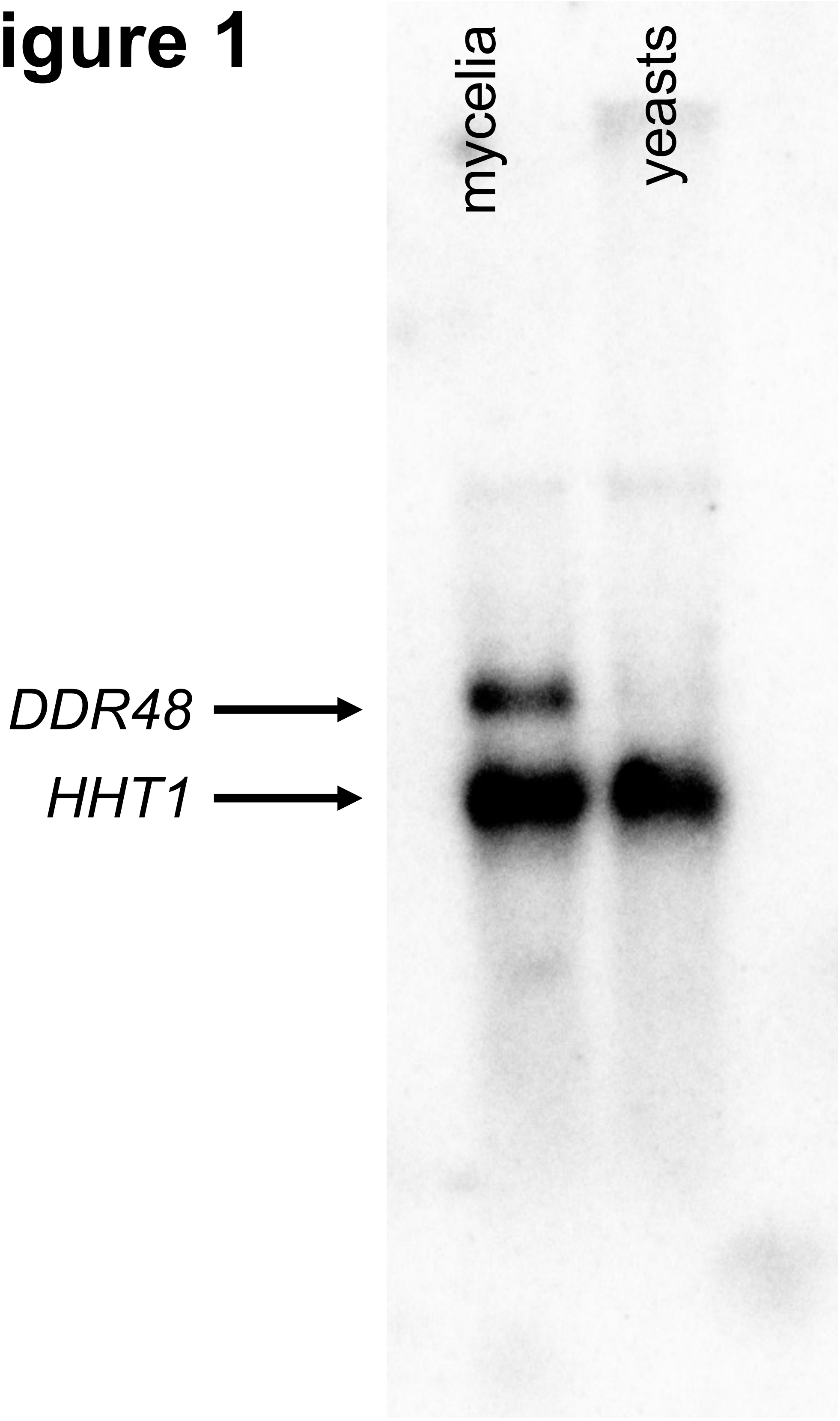

Figure S2

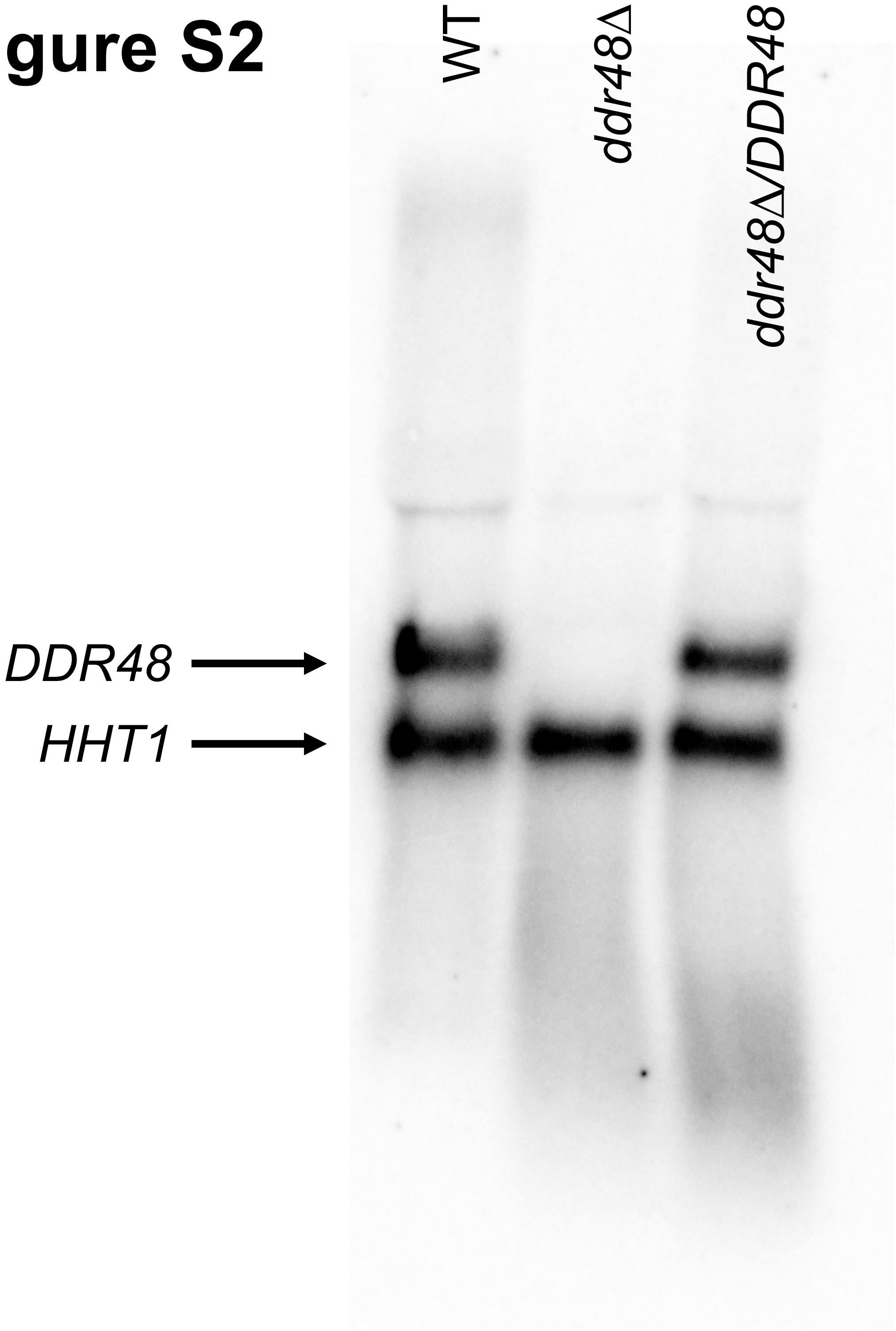

Supplement: Supplementary file 1 [file jof-07-00981-s001.zip › jof-1437875-supplementary.pdf]
